# Biology‐informed neural networks learn nonlinear representations from omics data to improve genomic prediction and biological discovery

**DOI:** 10.1111/tpj.71076

**Published:** 2026-08-11

**Authors:** Katiana Kontolati, Rini Jasmine Gladstone, Ian W. Davis, Ethan M. Pickering

**Affiliations:** ^1^ Bayer Crop Science St. Louis Missouri USA; ^2^ Department of Crop and Soil Sciences Institute of Crop Design, University of Georgia Athens Georgia USA

**Keywords:** Biologically‐informed neural networks, crop breeding, genomic prediction, genomic selection, genotype‐to‐phenotype modeling, interpretable deep learning, multi‐omics integration, pathway‐informed machine learning

## Abstract

Traditional genotype‐to‐phenotype models depend heavily on direct mappings that achieve only modest accuracy, forcing breeders to conduct large, costly field trials to maintain or marginally improve genetic gain. Models that incorporate intermediate molecular phenotypes can achieve higher predictive fit, but remain impractical since such data are unavailable at deployment or design time. Biology‐informed neural networks (BINNs) overcome this limitation by encoding pathway‐level inductive biases and leveraging multi‐omics data only during training, while using genotype data alone during inference. Here, we extend BINNs for genomic prediction and selection in crops by integrating thousands of single‐nucleotide polymorphisms with multi‐omics measurements and prior biological knowledge. By directly embedding omics‐derived priors, BINN outperforms conventional models in low‐data (*n* < *p*) regimes and enables sensitivity analyses that expose biologically meaningful traits. Applied to maize gene expression and multi‐environment field trial data, BINN improves rank correlation accuracy within and across most subpopulations under sparse data conditions and nonlinearly identifies genes that GWAS/transcriptome‐wide association studies may fail to uncover. With complete domain knowledge for a synthetic metabolomics benchmark, BINN substantially reduces prediction error relative to conventional neural nets and correctly identifies the most important nonlinear pathway. Importantly, both cases show that highly sensitive BINN latent variables correlate with the experimental quantities they represent, despite not being trained on them. This suggests that BINNs learn biologically relevant representations, nonlinear or linear, from genotype to phenotype. Together, BINNs establish a framework for improved genomic prediction accuracy and biological discovery that can guide genomic selection, candidate gene selection, pathway enrichment, and gene‐editing prioritization.

## Significance Statement

Genomic selection in plant breeding relies on genotypic data alone, while recent studies have shown that multi‐omics integration can boost prediction, yet such models remain impractical unless deployable without costly molecular measurements. Meanwhile, machine learning models hold great potential for capturing complex trait architectures but are largely uninterpretable, limiting biological insight. We address both challenges by embedding pathway knowledge into neural networks that learn transparent, omics‐consistent representations from genotypes alone making multi‐omics‐informed prediction practical for genomic selection while exposing the molecular mechanisms underlying complex crop traits.

## INTRODUCTION

Feeding 10 billion people by 2050 will require faster, more reliable crop improvement. In both conventional breeding—crossing and selecting offspring—and genome editing—making targeted edits and selecting edited lines—progress hinges on accurate genotype‐to‐phenotype (G2P) prediction to enable genomic selection (GS) (Crossa et al., 2017; Meuwissen et al., 2001). GS improves crop improvement efficiency by permitting early selection at the seed or edit stage and reducing dependence on slow, costly, multi‐location field trials (Heffner et al., 2009). With a well‐calibrated model trained on prior genotype–phenotype data, a single low‐cost genotyping assay can substantially reduce phenotyping requirements in both space and time (Crossa et al., 2017). A wide range of models has been applied to GS—including linear mixed models; machine‐learning methods such as random forests and kernel approaches; and deep neural networks—with performance varying by trait architecture, data volume, and environment (Breiman, 2001; Yegnanarayana, 2009). Current approaches fall short of capturing the true biological processes from genotype to phenotype, and the field has a long way to go toward accurate, mechanistically grounded prediction.

Despite the surge of countless AI models and architectures, linear mixed modeling approaches remain state of the art in G2P studies, with no other class of model consistently outperforming them across crops, populations, traits, years, and locations (Alemu et al., 2024; Azodi et al., 2019; Washburn et al., 2025). Although there are several adaptations to linear models, most are quite similar. For instance, ridge regression (McDonald, 2009) and its adaptation using best linear unbiased predictor, rrBLUP (Endelman, 2011), trained using genotypic (marker) data are identical, except rrBLUP chooses the regularization parameter, α, as a ratio of variances instead of by cross‐validation (Ogutu et al., 2012). Likewise a genomic BLUP or GBLUP (Clark & van der Werf, 2013) is mathematically equivalent to rrBLUP, but with compute efficiency gains when the number of lines is less than the number of markers (Jacquin et al., 2016), while the ‘Bayesian alphabet’ models (BayesA, BayesB, BayesC, etc.) (Gianola et al., 2009) differ in allowing different markers to have different priors (Montesinos López et al., 2022). Although several promising nonlinear artificial neural network (ANN) architectures have been proposed in the literature (Gao et al., 2023; Ma et al., 2017; Wang et al., 2025), deep learning methods have yet to show consistent improvements over traditional models and have not been adopted at scale in breeding pipelines (Montesinos‐López et al., 2021). This is partly due to over‐parameterization and the lack of tailored deep learning architectures. Further gains are unlikely without injecting biological structure such as pathways, interactions, and regulatory programs into the model, a role for which flexible AI architectures are well suited.

A natural route to inject such biological structure is to integrate intermediate multi‐omics signals—transcripts, proteins, metabolites—as scaffolds between genotype and phenotype, enriching G2P models with mechanistic constraints (Atwell et al., 2010; Azodi et al., 2020; Christensen et al., 2021; Heffner et al., 2009; Korte & Farlow, 2013; Meuwissen et al., 2001; Riedelsheimer et al., 2012). Approaches such as multi‐staged analysis (Ritchie et al., 2015) and transcriptome‐wide association studies (TWAS) (Gamazon et al., 2015; Wainberg et al., 2019) are proposed to integrate omics data, such as gene expression levels, between genotype and phenotype for association studies. Traditional linear mixed models, while capable of including additional covariates, are fundamentally limited in how they integrate multi‐omics information. In practice, they treat each data modality as a flat feature set and cannot impose the hierarchical, pathway‐level constraints that capture the flow from genotype through intermediate traits to phenotype. This lack of architectural flexibility prevents them from leveraging richer, layered representations, such as gene‐to‐metabolite cascades or expression‐driven subnetworks, that can substantially boost predictive power. In contrast, ANNs provide the flexibility to integrate multiple high‐dimensional data streams such as genomics, transcriptomics, proteomics, lipidomics, etc., for tasks ranging from regression and classification to unsupervised representation learning, a capability that traditional linear models lack (Poirion et al., 2021; Wang et al., 2021, 2023). In practice, downstream omics are often not available for novel genotypes at design or deployment, so they can only inform training—via priors, pretraining, or architectural constraints—rather than serve as inference‐time features.

Biologically informed neural networks (BINNs), also referred to as visible neural networks (VNNs) (Selby et al., 2025) embed omics data and domain‐specific knowledge, such as gene, protein, or pathway relationships, directly into their architecture to improve prediction accuracy, interpretability, and extrapolative potential. The concept of structuring neural network architectures around known biological hierarchies predates the BINN/VNN terminology itself. Lin et al. (2017) used biologically sparse networks for dimensionality reduction of single‐cell RNA‐Seq data, finding they outperformed fully connected models. Ma et al. (2018) then introduced DCell, a ‘visible neural network’ mapping Gene Ontology subsystems to neurons, enabling interpretable prediction of cell growth from genotype in yeast, widely regarded as a foundational work in this space (Selby et al., 2025). Around the same time, Chen et al. (2018) proposed GSAE, one of the earliest autoencoder‐based BINNs, while PASNet (Hao, Kim, Kim, & Kang, 2018) and Cox‐PASNet (Hao, Kim, Mallavarapu, et al., 2018) introduced pathway‐associated sparse networks for prognosis prediction and survival analysis. van Hilten et al. (2021) developed GenNet, mapping SNPs through gene and pathway layers with biologically constrained connectivity, though without using the term BINN. As Selby et al. (2025) note, terminology remains far from standardized: models have been described as ‘visible’, ‘knowledge‐primed’ (Fortelny & Bock, 2020), ‘knowledge‐guided’ (Lee & Kim, 2022), or ‘biologically informed’, complicating literature search and cross‐study comparison. The explicit BINN framing draws an analogy to physics‐informed neural networks (PINNs) that incorporate governing equations into their loss functions (Raissi et al., 2019). Where PINNs encode physical laws as soft constraints, BINNs impose biologically plausible sparsity patterns, reducing parameter count and data requirements while aligning latent representations with known biology, trading universal approximation for an inductive bias that generalizes better to specific biological systems (Miao, 2024; Selby et al., 2025).

Since these foundational works, BINNs have been applied broadly, with gene expression as the dominant omics modality and oncology as the primary application domain (Selby et al., 2025). Key applications include hierarchical pathway networks for prostate cancer discovery (P‐NET; Elmarakeby et al., 2021), drug response prediction integrating mutation data with drug chemical structure (DrugCell; Kuenzi et al., 2020), proteomics biomarker discovery (Hartman et al., 2023), multi‐omics prediction of complex traits (van Hilten et al., 2024), survival analysis (Cox‐PASNet; Hao, Kim, Mallavarapu, et al., 2018; PathExpSurv; Hou et al., 2023), and single‐cell transcriptomics via variational autoencoders such as VEGA (Seninge et al., 2021) and expiMap (Lotfollahi et al., 2023). Comparative studies have generally found that BINNs perform comparably to or better than fully connected networks of similar size while being significantly more sparse (Fortelny & Bock, 2020; Kuenzi et al., 2020), and that randomizing biologically informed connections leads to clear performance degradation (Pedersen et al., 2023). More recently, Nordenstorm et al. (2025) demonstrated that BINNs exhibit emergent self‐pruning: when false‐positive edges are deliberately introduced into the prior knowledge network, the model learns to assign them negligible weights during training under sufficient L2 regularization, suggesting that BINNs can tolerate uncertainty in prior knowledge without compromising mechanistic interpretability.

Despite their promise, several limitations persist. Existing BINNs typically enforce a rigid one‐to‐one mapping of neurons to biological entities, limiting architectural flexibility and nonlinear modeling capacity (Selby et al., 2025). Redundancy in pathway ontologies can lead to unstable explanations, mitigable via dropout (Fortelny & Bock, 2020) or pruning (ParsVNN; Huang et al., 2021). Robustness of pathway‐level interpretations remains a concern, as small sample sizes can cause significant variation across train–test splits or initializations (Esser‐Skala & Fortelny, 2023). The choice of pathway database such as Reactome, KEGG, Gene Ontology, or MSigDB can significantly influence results, yet few studies have systematically compared across databases (Selby et al., 2025). Nearly all VNN architectures rely on early fusion for multi‐omics data, which can result in loss of structural information (Hauptmann & Kramer, 2023; Pedersen et al., 2023). Most existing BINNs still depend on omics measurements at deployment, undermining their practicality in plants and crop design. Furthermore, their method for ranking biological entities typically relies on learned single‐edge weights—conceptually similar to GWAS or TWAS—and consequently tends to highlight already known significant genes while overlooking those involved in nonlinear or epistatic interactions. Finally, the potential for BINNs to discover genuinely novel biological relationships through pathway expansion (Hou et al., 2023) or neural architecture search remains largely untapped (Selby et al., 2025).

In this work, we develop a BINN framework for applications in agriculture, specifically genomic prediction, selection and breeding, and the discovery of intermediate molecular traits. Although BINNs have shown considerable promise in biomedical and clinical settings, no study to date has adapted or deployed such architectures for plant genomic prediction. Agriculture presents a distinct set of challenges: models must handle high marker density and complex population structure, remain interpretable for breeders seeking to understand the biological basis of trait variation, and critically must not require omics measurements at deployment, so that they remain practical for large‐scale breeding programs where only genotypic data are routinely available.

**Figure 1:**
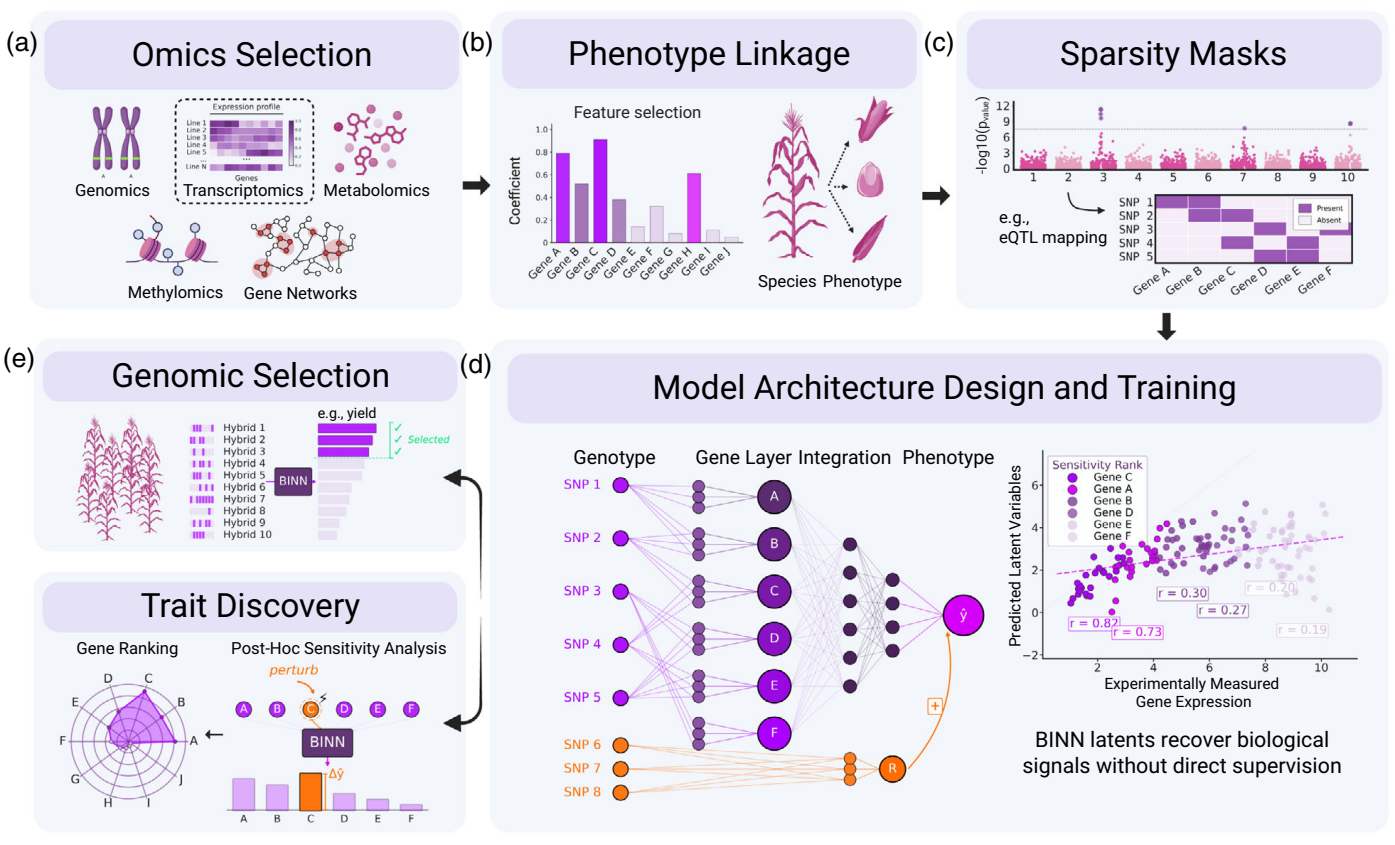
Biology‐informed neural networks embed domain knowledge for enhanced genomic prediction and learning nonlinear biological relationships. (a) One or more omics modalities (e.g., transcriptomics, metabolomics) that hold predictive power for the target trait are selected as the biological foundation for model design. (b) Omics features are linked to the phenotype to perform a biologically guided pre‐selection of relevant features (e.g., genes associated with the trait of interest). (c) Selected omics features are mapped back to genotypic variation (e.g., via eQTL mapping), establishing the link between SNP markers and the molecular features that drive phenotypic variation. (d) These mappings are used to construct a biologically informed neural network architecture, where network layers and connectivity reflect known biological relationships. Notably, latent variables learned by the model correlate with experimentally measured omics values, demonstrating that the network captures biologically meaningful representations without requiring omics data as input at deployment. (e) The trained model enables two key applications: (i) genomic selection, where phenotypic predictions are made from genotypic data alone for use in breeding pipelines and (ii) trait discovery, where model interpretation identifies candidate molecular traits and mechanisms underlying complex traits. Created with BioRender.com.

To address these challenges, we propose a flexible BINN architecture that can seamlessly ingest multiple omics layers, mapping information from one biological level to the next, while inserting fully connected hidden layers within each biological module to improve expressivity. Rather than exhaustively encoding the entire known interactome—an approach that would demand far more data than is typically available in plant breeding—the model begins with a preselected set of intermediate molecular traits (e.g., genes identified through a data‐driven screening step) that are most likely to be phenotype‐relevant, thereby focusing the network's capacity on the biological pathways that matter the most. While conceptually related to DeepHisCoM (Park et al., 2022) and DeepBINN (Meirer et al., 2024), which model each pathway as a multi‐layer dense sub‐network and read out pathway importance from a final single‐node layer, our framework preserves layered nonlinearity throughout the full depth of the model rather than condensing biological attribution into last‐layer scalar weights. This balance between expressivity and biological transparency is central to the design: the fully connected subnetworks within each biological module enable the model to capture complex nonlinear interactions among molecular features, while the sparse, biologically structured connectivity between modules preserves pathway‐level organization aligned with known biology, enabling *post hoc* discovery of which pathways contribute most to the phenotype (Figure 1).

We demonstrate that prediction accuracy remains comparable to, or exceeds, that of established G2P mixed linear models widely used in the plant breeding literature. Notably, the BINN latent variables identified as most sensitive by the framework exhibit strong correlations with their corresponding experimentally measured quantities, even though these measurements were never used as training targets. This suggests that the framework learns biologically relevant representations along the path from genotype to phenotype. Furthermore, through a *post hoc* sensitivity analysis, the framework enables targeted pathway prioritization and ranks intermediate molecular traits that lie beyond the reach of conventional GWAS and TWAS approaches. In addition, we show the flexibility of incorporating omics information directly into the loss function as soft constraints to avoid over‐constraining the model given the inherently dynamic and noisy nature of molecular phenotypes. We further demonstrate the ability to transfer across related populations, maintaining strong prediction accuracy when genetic backgrounds are only partially shared. Importantly, our framework decouples training from deployment: multi‐omics data inform model training, but only genotypic information is required at inference time, making it a practical and scalable tool for crop GS and design.

## RESULTS

We develop and evaluate BINN models informed by two distinct types of intermediate omics—*transcriptomics* and *metabolomics*—to predict phenotypes directly from genotype. Our first case study leverages transcriptomic profiles measured in a large‐scale maize field experiment (Torres‐Rodríguez et al., 2024), where lines from multiple heterotic groups were grown under real agronomic conditions. This setting provides a realistic testbed for BINNs: gene expression captures environmentally modulated regulatory activity, offering a richer signal than raw marker data. In this context, we observe not only promising gains over conventional G2P models but also important caveats due to the noise, heterogeneity, and incomplete pathway knowledge that naturally accompany field‐derived omics measurements. In contrast, our second case study is a synthetic benchmark based on metabolomic traits simulated through an ordinary differential equation (ODE) model of shoot branching (Bertheloot et al., 2020). Unlike the maize field data, this framework provides ‘perfect’ domain knowledge of the true causal pathways, enabling us to rigorously stress‐test BINNs and evaluate their ability to recover mechanistic structure when the ground truth is fully known. Together, these complementary case studies—one grounded in realistic breeding data, the other in controlled simulated biology—allow us to assess both the practical utility and the methodological limits of BINNs. Table 1 summarizes implementation details; Full configurations and preprocessing steps can be found in the Supporting Information.

**Table 1:** Implementation components for the maize TWAS dataset and synthetic shoot‐branching case studies

Attribute	Maize TWAS dataset	Synthetic shoot‐branching
Input SNPs/genes	∼20 000 SNPs per trait	1600 genes
Intermediate omics data	Transcriptomics	Metabolomics
# Intermediate traits	∼1000 genes per trait	4 (auxin, sucrose, cytokinin, strigolactone)
# Output phenotypes	4 (days to anthesis NE, MI; days to silking NE, MI)	1 (time to bud outgrowth)
Trait selection method	ElasticNet regression	From known ODE model
SNP mask construction	eQTL mapping of selected genes	ODE‐derived gene–metabolite mappings
Loss function	Mean squared error (MSE)	Soft‐constrained MSE
Reference	Torres‐Rodríguez et al. (2024)	Bertheloot et al. (2020) and Powell et al. (2022)

### Transcriptomics‐derived BINN

#### Gene‐expression‐informed BINNs improve Spearman rank correlations over G2P models across and within most populations in maize field trials

**Figure 2:**
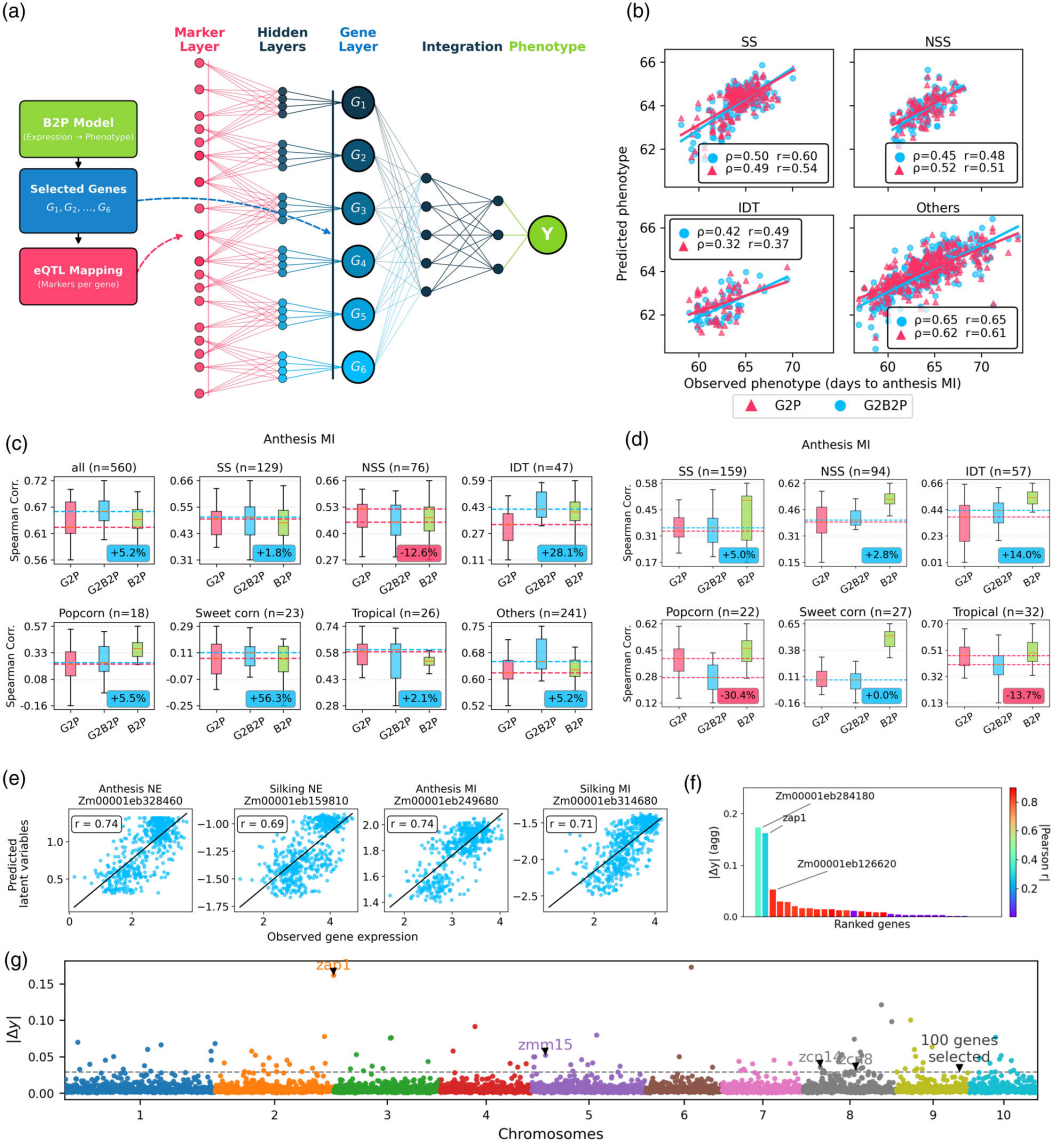
Biology‐informed neural networks (BINNs) improve prediction accuracy and biological discovery through utilization of gene expression in genotype to phenotype modeling. (a) Schematic of the transcriptomics‐derived BINN architecture. The SNP marker and gene expression data are utilized for feature selection, which serves to sparsify the connections between the input and intermediate layers of the BINN architecture. The marker data for each gene are passed through pathway subnetworks at the intermediate layer and the outputs from this layer are passed through a non‐linear integrator network to predict the phenotype values. The G2P and B2P models are both linear models. (b) Predicted versus observed phenotype—days to anthesis in MI—across four subpopulations (SS, NSS, IDT, and Others). Points show G2P (GBLUP on genotype; pink triangles) and G2B2P (BINN with expression‐informed sparsity; blue circles). An ordinary least‐squares (OLS) fit is overlaid and the Spearman correlation (ρ) is reported in the legend. (c) Test Spearman correlation distributions for days to anthesis in MI, comparing three predictive models (G2P, G2B2P, and B2P: Ridge regression on expression, green) across five independent 20%/80% train/test splits, each with fivefold cross‐validation to capture both split‐level and CV‐level variability. Each subplot shows results for all lines pooled (‘all’) and for each of the seven distinct subpopulations (SS, NSS, IDT, Popcorn, Sweet corn, Tropical, and Others). The pink dashed line marks the median of the G2P baseline; the dashed line for G2B2P is colored green when its median exceeds that baseline or red when it falls below. Legends report the median percent change of G2B2P relative to G2P and titles show the number of test samples. (d) Test Spearman correlation distributions for leave‐one‐population‐out experiments for days to anthesis in MI, comparing the same three predictive models. Each subplot shows results for the predictions on six distinct subpopulations using the models that are trained by leaving out the corresponding subpopulation from the training data. (e) Predicted latent variables versus observed gene expression for four representative high‐correlation genes per phenotype. (f) Aggregated absolute phenotypic change (|Δ*y*|) for representative genes (15 highest and 15 lowest mean |Pearson *r*|, plus top‐2 by sensitivity), sorted by sensitivity. Bar color indicates per‐model mean |*r*| between predicted latent variable and observed gene expression. (g) Aggregated absolute phenotypic change per perturbed gene, with a threshold capturing the BINN‐derived 100 most significant genes, including zap1, zmm15, zcn14, and zcn8.

BINNs embed expression‐derived sparsity into a genotype‐to‐biological‐knowledge‐to‐phenotype ‘G2B2P’, where biological knowledge is gene expression data, network which delivers superior predictive performance compared to genotype‐only GBLUP models, under sparse data conditions (Figure 2b–d). We use the maize TWAS dataset from Torres‐Rodríguez et al. (2024), which provides matched genotypes, RNA‐seq expression profiles, and flowering‐time phenotypes: days‐to‐anthesis and days‐to‐silking in Nebraska and Michigan. In five independent 20%/80% train/test splits with fivefold cross‐validation on each training set, BINN achieves higher Spearman correlations than the GBLUP baseline, both when pooling all 693 lines and within most of the seven heterotic subpopulations, though gains are not uniform—for example, BINN shows clear improvements in SS, IDT, and others but not consistently in NSS (Figure 2b,c). Across all pairwise comparisons, BINNs outperformed GBLUP in the majority of cases, and the overall advantage was statistically significant on a paired *t*‐test ($p=2.23\times 10^{-6}$). Here, we report representative results for anthesis MI as shown in Figure 2c; complete results for all phenotypes are provided in Figure S5. Notably, the most pronounced and consistent improvements occurred within individual subpopulations, the scenario in which GS is applied commercially, demonstrating BINN's ability to capture subtle, group‐specific genetic variation.

#### BINNs leverage domain knowledge to pinpoint key genes and derive SNP–gene connections that both shrink network size while preserving nonlinear modeling power

Models that embed domain knowledge have frequently demonstrated similar or superior predictive power compared to purely genotype‐based approaches (Azodi et al., 2020). In the maize TWAS application, we observe that expression‐based domain‐to‐phenotype (B2P) models outperform conventional G2P baselines across most evaluation settings and BINNs can translate this predictive strength into the G2P setting. Transcriptomic profiles inform the degree of architectural sparsity without ever feeding raw expression values into the prediction network at inference. Specifically, we first fit an Elastic Net model to each flowering‐time trait—tuning its L1/L2 penalty via cross‐validation—and selected the genes with nonzero coefficients, noting that the feature selection outcome was particularly sensitive to the specific cross‐validation split. We then carried out eQTL mapping on these candidates to nominate their top SNP markers, which defined the sparse SNP → gene mask that shapes our G2B2P BINN architecture (Figure 2a). As shown in Table 2, a sweep of the Elastic Net's L1 ratio revealed that retaining approximately 1000 genes per trait and split offers the best trade‐off between model compactness and accuracy, recovering the major regulators identified in the original study.

**Table 2:** Test set Spearman rank correlation of BINNs across all populations and cross‐validation splits for various Elastic Net L1 ratios used to select genes for the G2B2P mask for all four phenotypes

L1 ratio	# genes	Anthesis NE	Anthesis MI	Silking NE	Silking MI
0.05	1541–2251	0.650 ± 0.034	0.652 ± 0.046	0.630 ± 0.041	0.649 ± 0.036
**0.10**	**848–1271**	**0.655** ± **0.037**	**0.663** ± **0.034**	**0.632** ± **0.040**	**0.660** ± **0.044**
0.20	467–696	0.605 ± 0.151	0.656 ± 0.038	0.623 ± 0.043	0.662 ± 0.038
0.50	132–301	0.595 ± 0.143	0.633 ± 0.053	0.593 ± 0.127	0.640 ± 0.040
1.00	50–131	0.597 ± 0.047	0.596 ± 0.047	0.547 ± 0.114	0.593 ± 0.059

The best setting for each trait is bolded.

#### BINNs exploit shared expression–trait mechanisms beyond marker structure to improve generalization but rigid priors can limit flexibility in genetically distant populations

It is well established that marker data are strongly tied to population structure whereas expression data are often tissue and environment‐specific and less tightly tied to ancestry (Torres‐Rodríguez et al., 2025). Removing systematically distinct populations from the BINN training set revealed a clear pattern in model transferability. Expression data overall offer improved cross‐population generalization compared to marker‐based models as expression reflects functional output of many regulatory layers that can ‘normalize’ some of the divergence in raw markers. However, BINN's biologically constrained architecture can only capitalize on this advantage when the causal paths through biology are shared between train and test lines. Evidently, when one of the mainstream heterotic groups such as SS, NSS, or IDT was held out, BINN maintained robust generalization performance (see Figure 2d). This is particularly important for large‐scale breeding programs, which rely heavily on these temperate pools for developing elite germplasm and driving genetic gain. In contrast, when genetically more distant populations were excluded from training, such as sweet corn or tropical, which represent cases where regulatory programs diverge substantially from temperate pools (Wu et al., 2015), BINN appeared to overfit to pathway patterns dominated by the larger heterotic groups thereby limiting its ability to adapt. Thus, while BINNs excel when shared biological mechanisms exist, overly rigid priors limit their flexibility in zero‐shot learning scenarios. However, this issue diminishes as domain knowledge improves: the more precise and mechanistically grounded it is, the larger the performance gains across tasks (see Metabolomics‐derived BINN section).

#### Sensitivity analysis reveals nonlinear genetic contributors that TWAS/GWAS may fail to capture

A key advantage of BINNs lies in their capacity for biological discovery, that is, the ability of trained models to elucidate how specific biological entities influence intermediate traits and ultimately shape the phenotype. Traditional BINNs with single‐edge weights offer direct parameter‐level attribution, as each weight corresponds to a distinct marker–gene or gene–trait connection. In our implementation, we enhance model expressivity by replacing these single‐edge weights with fully connected layers, sacrificing one‐to‐one parameter attribution but enabling richer representations of nonlinear dependencies. To assess whether BINNs can identify biologically meaningful genes, we developed and conducted a sensitivity analysis across all pathway subnetworks. Specifically, we perturbed each pathway's latent variable in both directions and quantified the resulting change in the predicted phenotype, ranking genes by their aggregated effect. Figure 2g summarizes this analysis, showing that BINN recovers several TWAS‐significant genes reported in Torres‐Rodríguez et al. (2024), such as the previously characterized zcn8 as well as zap1, zmm15, and zcn14, but also highlights additional candidates that may participate in epistatic interactions shaping phenotypic response. These genes may be overlooked by traditional approaches like TWAS, which rely on marginal gene–trait associations, whereas BINN's end‐to‐end training captures predictive, nonlinear, and combinatorial effects beyond the reach of standard linear models. Interestingly, many genes with high sensitivity to phenotype perturbations also exhibit moderate‐to‐strong latent‐expression correlations (Figure 2e,f), suggesting that BINNs preferentially learn biologically grounded representations aligned with known mechanisms. However, Figure 2f also reveals that this relationship is not universal: notably, zap1—the second most sensitive gene, consistent with its prominence in the Manhattan plot (Figure 2g)—exhibits low mean |r|. While latent‐expression correlations provide useful quasi‐validation of the learned representations, sensitivity scores offer a more reliable measure of gene importance.

### Metabolomics‐derived BINN

We now turn to a fully specified simulation where the underlying biological system is known, allowing us to evaluate not only prediction accuracy but also what the BINN learns internally and how it distributes representational capacity across pathways. This contrasts with the previous example, which relied solely on real‐world experimental data. To illustrate, we use a synthetic metabolomics problem in which hormone–sugar interactions are formalized through an ODE model, creating a clean G2P nonlinear test bed for evaluating BINNs. A motivating example comes from axillary bud outgrowth—the switch‐like process underlying shoot branching—arising from interactions among auxin (A), cytokinin (CK), strigolactone (SL), and sucrose (S) in a minimal network (Bertheloot et al., 2020), later extended with genotype‐to‐metabolite variation at multiple causal loci (Powell et al., 2022). The domain knowledge we embed is simple but definitive: gene–metabolite, metabolite–metabolite, and metabolite–phenotype interactions. Importantly, the BINN is not provided with the full ODE details (the magnitudes of effects, equation structures, or nonlinearities) but only the associations between layers. To avoid an artificially ‘easy’ setting and to test robustness, we perturb the simulated data by adding 5% noise to intermediate traits and 10% noise to phenotypes. This controlled setting enables us to rigorously test the BINN methodology: evaluating performance under both plentiful and sparse data conditions, examining its ability to recover known causal dynamics, and exploring extensions such as custom loss functions applied to partially observed intermediates.

#### BINNs improve predictions under sparse data regimes

**Figure 3:**
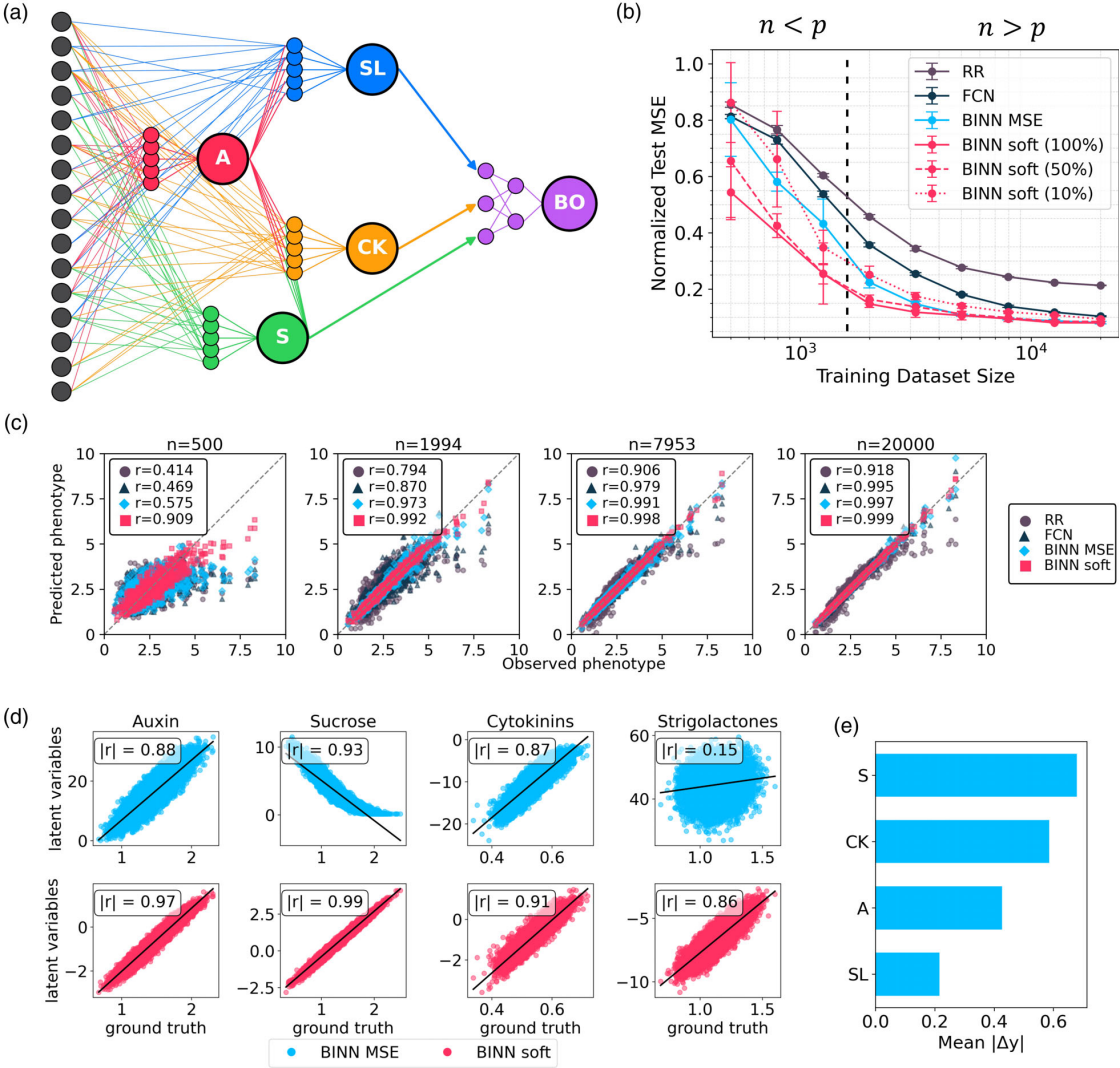
Biology‐informed neural networks (BINNs) significantly outperform baselines in the sparse data limit n<p. (a) Schematic of the BINN architecture for the shoot‐branching network. Gene inputs are routed through four biologically annotated pathway subnetworks corresponding to auxin (A), sucrose (S), cytokinins (CK), and strigolactones (SL), and then combined in a final integrator to predict bud‐outgrowth time (BO). (b) Test‐set performance (MSE) for six models: ridge regression (RR), a generic fully connected network (FCN), BINN trained with standard MSE (BINN‐MSE), and BINN trained with the biologically informed soft‐constraint loss (BINN‐soft) with a varying fraction of known intermediate trait measurements (100, 50 and 10%), evaluated across nine training set sizes logarithmically spaced between 500 and 20 000 samples. Boxplots display the distribution across five random initializations. The vertical black dashed line at n=1600 denotes the transition from sparse (n<p) to plentiful (n>p) data regimes. (c) Predicted versus observed phenotype across four training sizes (n = 500, 1994, 7953, 20 000) for four models: RR (purple circles), FCN (black triangles), BINN MSE (blue diamonds), and BINN soft (red squares). Each panel shows the identity line (*y* = *x*), and in‐panel legends report Pearson correlation (*r*) for each model. (d) Scatter plots of predicted versus true latent values for each intermediate trait (A, S, CK, SL) under the BINN MSE (blue) and the BINN soft model (red) trained with *n* = 1994 samples. The fitted regression line and absolute Pearson correlation coefficient, |*r*|, are overlaid. (e) Aggregated absolute phenotypic change per perturbed intermediate trait for the BINN MSE model trained with *n* = 1994 samples.

In the genotype‐to‐metabolites‐to‐phenotype setup, BINN embeds ODE‐derived metabolite pathways and constrains gene inputs through known gene–metabolite links (Figure 3a). Evaluated across nine geometrically spaced training sizes from 500 to 20 000 lines, with five random splits, the standard MSE‐trained BINN (BINN MSE) consistently outperformed ridge regression (RR) and an unconstrained fully connected network (FCN) in the sparse data regime, that is, when the number of training lines n was smaller than the input dimensionality p (Figure 3b,c). As expected, when sample size increases, BINN performance approaches that of the FCN, indicating that the pathway‐guided sparsity yields a better bias–variance trade‐off when data are limited, while also preserving nonlinear expressivity as data grow. This demonstrates that BINNs can faithfully capture the intrinsic nonlinearity encoded in the underlying ODE dynamics across the entire data range, an aspect fundamentally beyond the reach of linear models like RR and GBLUP in the data‐plentiful limit (n≫p) and common neural networks in the data‐sparse limit (n≪p).

#### Biology‐informed loss functions, applied to intermediate variables, improve predictive ability and recovery of pathway dynamics with sparse intermediate labels

In many systems, intermediate traits can be experimentally measured, and this information can be used to better anchor latent variables to pathway dynamics. To this end, we introduce a soft‐constraint loss function (i.e., Pearson loss) that explicitly encourages latent outputs to correlate with ground‐truth intermediate trait values. In realistic scenarios, budget or experimental constraints often limit the amount of intermediate data that can be collected. To reflect this, we evaluate the model under three conditions: 100, 50, and 10% of lines with known labels. This design preserves genotype‐only inference while allowing for complete or partial pathway supervision during training. The soft‐constraint approach (BINN soft) achieves the lowest test MSE in the small‐data regime and performs comparably to standard BINNs with larger datasets (Figure 3b). Notably, reducing the available intermediate data from 100 to 50% has minimal impact on performance, demonstrating strong robustness of the BINN soft model to partial supervision. However, when only 10% of intermediate trait information is retained, the performance of BINN soft converges with that of the BINN MSE model, which receives no intermediate supervision at all, suggesting that there exists a minimum threshold of biological annotation below which the soft‐constraint signal becomes too sparse to meaningfully guide latent representations.

#### BINN architectures provide a latent variable mechanism for biological discovery, uncovering the critical importance of upstream pathways in determining bud outgrowth

When we analyze the biologically informed latent variables via scatter plot diagnostics on 20 000 held‐out test lines, distinct pathway‐level patterns emerge. We ran this experiment under both the standard MSE objective (BINN MSE) and a custom soft‐constraint MSE (BINN soft). As expected, the soft‐constraint setup (see Figure 3d, second row) yields strong correlations between latent variables and ground‐truth metabolites across all pathways, since the model was partially supervised to do so. However, the more revealing insight comes from Figure 3d (first row): under the standard BINN MSE, which receives no intermediate supervision whatsoever, the model recovers strong correlations with ground‐truth auxin (|*r*| = 0.88), cytokinins (|*r*| = 0.87), and sucrose (|*r*| = 0.93), while strigolactones exhibit only a weak association (|*r*| = 0.15). Recent work has shown that architectural transparency alone does not guarantee identifiability of internal representations (Caranzano et al., 2026); many parameterizations can produce the same input–output function. Our eQTL‐derived masks break the strongest source of nonidentifiability—permutation symmetry—ensuring the right information flows through the right node, but do not constrain sign or scale within each node. Accordingly, we do not interpret latent variable values as biological measurements, and the sign of the correlation is not meaningful in this context. However, we found that even though the sign flips randomly across random seeds, the magnitude |r| remains stable, indicating that the mutual information between latent nodes and true biology is an invariant of the learned function. Importantly, this is consistent with the *post hoc* sensitivity analysis in Figure 3e, which ranks sucrose and cytokinins as the most influential intermediates, followed by auxin, with strigolactones contributing comparatively little. This ranking aligns with a variance decomposition of the data‐generating process: Sucrose dominates phenotypic variance through its wide range and strong nonlinear gating effect, cytokinins contribute via a steep inhibitory function, and strigolactones operate in a saturated regime where variation has a smaller downstream impact. The agreement between sensitivity rankings and ground‐truth variance structure confirms that the BINN architecture recovers meaningful biological structure even without explicit intermediate supervision. These results illustrate how BINNs can learn nonlinear relationships and surface biologically meaningful variables when appropriate inductive bias is provided, enabling pathway discovery through both explicit alignment losses (BINN soft) and emergent signals validated via sensitivity analysis in the unconstrained setting (BINN MSE).

## DISCUSSION

In this work, we benchmarked biologically informed neural networks on two complementary tasks: a real maize TWAS dataset (Torres‐Rodríguez et al., 2024) and an ODE‐grounded synthetic shoot‐branching problem (Bertheloot et al., 2020; Powell et al., 2022) to test whether they deliver accurate, deployable, and biologically transparent genomic predictions. Across both applications, BINNs outperformed baseline GBLUP, RR, and FCNs in the sparse data regime, remained stable under noise, and required only genotypes at inference.

Although multi‐omics data carry strong predictive power for complex traits (Azodi et al., 2020; Christensen et al., 2021; Riedelsheimer et al., 2012), such measurements are typically unavailable for novel genotypes at design or deployment time, limiting their direct use in GS pipelines (Crossa et al., 2017; Heffner et al., 2009). Existing BINN implementations have focused primarily on biomedical applications where omics inputs are available at inference (Elmarakeby et al., 2021; Kuenzi et al., 2020; Selby et al., 2025). Our framework addresses this gap by translating omics information into architectural sparsity and optional soft‐constraint losses during training, while requiring only genotypic data at inference, maintaining full compatibility with standard GS workflows where a single low‐cost genotyping assay drives selection decisions (Crossa et al., 2017).

Linear mixed models remain the state of the art for G2P prediction, with deep learning methods yet to show consistent improvements, partly due to over‐parameterization (Alemu et al., 2024; Azodi et al., 2019; Montesinos‐López et al., 2021; Washburn et al., 2025). BINNs address this by deriving sparse connectivity masks from omics‐guided feature selection and association analyses, reducing effective parameter count to match trait complexity. Unlike existing BINNs that enforce rigid one‐to‐one neuron‐to‐entity mappings (Ma et al., 2018; Selby et al., 2025), we employ fully connected subnetworks within each pathway module—conceptually related to DeepHisCoM (Park et al., 2022) and DeepBINN (Meirer et al., 2024) but preserving layered nonlinearity throughout the full model depth rather than condensing interpretation into last‐layer scalar weights. This design enables each module to capture intra‐pathway epistasis while the final integrator recovers cross‐pathway interactions. The tuned models achieved improved performance relative to G2P baselines, with gains concentrated in the sparse data regime.

A key finding is that BINN latent variables correlate strongly with experimentally measured intermediate traits even without explicit supervision, suggesting the model preferentially allocates representational capacity to the most phenotype‐relevant pathways. This emergent alignment is consistent with comparative studies showing that biologically constrained connectivity guides learning toward mechanistically relevant features (Fortelny & Bock, 2020; Hartman et al., 2023). Through *post hoc* sensitivity analysis, BINNs recovered known TWAS‐significant genes such as *zcn8*, *zap1*, *zmm15*, and *zcn14* (Torres‐Rodríguez et al., 2024) while also highlighting additional candidates potentially involved in epistatic interactions that marginal association approaches like TWAS would miss (Gamazon et al., 2015; Wainberg et al., 2019). These results position BINNs as a complementary tool for candidate gene prioritization in functional genomics and gene‐editing applications.

Expression data are less tightly tied to population structure than marker data (Torres‐Rodríguez et al., 2025), and BINNs maintained robust generalization when mainstream heterotic groups (SS, NSS, IDT) were held out. However, for genetically distant populations such as sweet corn and tropical lines, where regulatory programs diverge substantially (Wu et al., 2015), the biologically constrained architecture has limited adaptability. While BINNs can tolerate some uncertainty in prior knowledge through emergent self‐pruning (Nordenstorm et al., 2025), overly rigid priors reduce flexibility in zero‐shot scenarios, a limitation that diminishes as domain knowledge improves.

Several practical considerations apply. The degree of sparsity must be carefully calibrated, as too little or too much undermines performance, and omics datasets are often noisy and heterogeneous, risking architectural mis‐specification (Esser‐Skala & Fortelny, 2023; Selby et al., 2025). Biologically informed soft‐constraint losses improved accuracy in the metabolomics setting but not for the transcriptomics dataset, and hard‐constraint formulations reduced flexibility, highlighting that the benefit of auxiliary supervision is context‐dependent. More generally, constraints can be implemented at various levels of stringency—from simply enforcing non‐negative latents (breaking sign symmetry) to requiring rank‐order agreement with measured intermediates, to penalizing correlation deviations, to fully matching measured values—each progressively resolving additional sources of nonidentifiability in the latent space (Caranzano et al., 2026). However, stronger constraints also limit the network's representational flexibility: hard‐constraint formulations reduced predictive performance in our experiments, and even soft penalties introduce a tradeoff between biological fidelity of the latent space and phenotype prediction accuracy that must be carefully tuned. More broadly, BINN gains depend critically on the quality of embedded domain knowledge: purely data‐driven setups are constrained by the noise and context dependence of experimental measurements, whereas sharper mechanistic knowledge yields markedly larger improvements.

Systematic benchmarking across a broader spectrum of traits from highly heritable to complex polygenic attributes and diverse crop species will be essential. Future work should explore integrating environmental covariates, additional omics layers (Table 3), and alternative topologies such as parallel‐layer BINNs (Hauptmann & Kramer, 2023; Pedersen et al., 2023). Computational advances including genomic language models (Benegas et al., 2023; Benegas, Albors, et al., 2025; Benegas, Ye, et al., 2025; Nguyen, Poli, Durrant, et al., 2024; Nguyen, Poli, Faizi, et al., 2024), sequence‐to‐function models that predict molecular phenotypes directly from DNA sequence such as Enformer (Avsec et al., 2021), SpliceAI (Jaganathan et al., 2019), and AlphaGenome (Avsec et al., 2026), and variant effect predictors that score the functional impact of individual mutations genome‐wide (Frazer et al., 2021) can convert provisional gene annotations into stronger inductive biases. For instance, sequence‐to‐function models, as they continue to improve, could generate *in silico* expression or chromatin‐accessibility profiles for any genotype, providing the intermediate molecular phenotypes that BINNs currently derive from experimental omics but at a fraction of the cost and without tissue‐ or environment‐specific sampling constraints. As biological knowledge deepens and computational tools for functional annotation mature, BINNs are well positioned to serve as the connective architecture between genomic variation and breeding decisions, delivering genotype‐only predictors that are not only accurate but biologically transparent enough to guide selection, pathway prioritization, and gene‐editing strategies at scale.

**Table 3:** Biological layers, their functions, and example data types

Biological level	Function	Example data type
Genotype	Genetic information underlying traits	SNP arrays, whole‐genome sequencing
Epigenetic modifications	Influence gene regulation without changing DNA sequence	DNA methylation
Gene expression	Controls gene activity affecting traits	RNA‐seq
Protein interaction networks	Provide structural and signaling connections influencing outcomes	Proteomics
Metabolites	Biochemical traits linking genes to observable traits	Metabolomics
Regulatory pathways	Control and coordinate gene expression	Pathway databases (e.g., KEGG)
Phenotype	Observable traits resulting from genetic and environmental interactions	Yield, height, flowering time, etc.

## METHODS

Here, we outline the BINN design used throughout before introducing the formalism. Our networks preserve nonlinear flexibility while enforcing biology‐derived sparsity: genotype inputs are routed through pathway modules defined by masks built from prior knowledge (e.g., feature selection, association/eQTL hits, curated pathways, or ODE‐based links). Each module is a small fully connected subnetwork that can model local nonadditivity and epistasis, and the module latents are fused by a final integrator to predict the phenotype. Intermediate omics measurements (e.g., expression, metabolites) inform the masks and, when available, can weakly supervise the latents via a soft constraint during training, but are never required at inference, preserving genotype‐only deployment. The same template flexibly instantiates single‐layer, stacked, staggered, or parallel multi‐omics variants (see Supporting Information) without changing the mathematical core.

### Model development with biological inductive biases

We consider n samples with p SNP features collected in the matrix $X\in {\mathbb{R}}^{n\times p}$ and a scalar phenotype vector $y\in {\mathbb{R}}^{n}$. Our BINN embeds L sequential omics layers between X and y, each layer l producing a latent representation $U^{\left(l\right)}\in {\mathbb{R}}^{n\times k_{l}}$ from its input $U^{\left(l-1\right)}$. We set $U^{\left(0\right)}=X$, $k_{0}=p$ and denote the number of subnetworks, corresponding to biological entities such as genes, metabolites, or proteins, at layer l by $k_{l}$.

#### Pathway subnetworks

At omics layer l, prior biological knowledge (e.g. eQTL links, pathway membership) is encoded by a binary mask $M^{\left(l\right)}\in {\left\{0,1\right\}}^{k_{l-1}\times k_{l}}$. Specifically,

$$M_{\mathrm{ij}}^{\left(l\right)}=\left\{\begin{array}{ll} 1, & \text{if feature}\;i\;\text{of}\;U^{\left(l-1\right)}\;\text{maps to entity}\;j\;\mathrm{at}\;\text{layer}\;l,\\ 0, & \text{otherwise}. \end{array}\right.$$

For each of the $k_{l}$ entities, we define a subnetwork $f_{j}^{\left(l\right)}$ that processes only the selected inputs $U^{\left(l-1\right)}M_{:,j}^{\left(l\right)}$. These subnetworks may have multiple hidden layers with sigmoid activations $\sigma \left(u\right)=1/\left(1+e^{-u}\right)$ to capture Hill‐type response typical in biological systems. We then concatenate their outputs into

$$T^{\left(l\right)}=\left[f_{1}^{\left(l\right)}\left(U^{\left(l-1\right)}M_{:,1}^{\left(l\right)}\right),\ldots ,f_{k_{l}}^{\left(l\right)}\left(U^{\left(l-1\right)}M_{:,k_{l}}^{\left(l\right)}\right)\right]\in {\mathbb{R}}^{n\times k_{l}},$$

and set $U^{\left(l\right)}=T^{\left(l\right)}$.

#### Residual network

To capture genetic effects not explained by any pathway subnetworks, we apply an unconstrained residual network $g_{r}$ (with parameters $\theta_{r}$) to the subset of SNPs that are not connected in any mask $M^{\left(l\right)}$. Denoting this unannotated SNP matrix by $X_{\mathrm{res}}\subset X$, the residual network directly predicts a scalar residual phenotype:

$$r=g_{r}\left(X_{\mathrm{res}};\theta_{r}\right)\in {\mathbb{R}}^{n},$$

ensuring that r captures variation attributable to SNPs outside known biological pathways.

#### Final integrator network

After the last omics layer produces $U^{\left(L\right)}\in {\mathbb{R}}^{n\times k_{L}}$, we concatenate it with the residual output $r\in {\mathbb{R}}^{n\times 1}$:

$$Z=\left[\;U^{\left(L\right)},r\;\right]\in {\mathbb{R}}^{n\times \left(k_{L}+1\right)}.$$

A final feed‐forward network h with parameters $\theta_{f}$ then maps Z to the predicted phenotype

$$\hat{y}=h\left(Z;\theta_{f}\right)\in {\mathbb{R}}^{n}.$$

which allows for the recovery of potential cross‐pathway interactions.

All network parameters ${\left\{W^{\left(l\right)},b^{\left(l\right)}\right\}}_{l=1}^{L}$, $\theta_{r}$, and $\theta_{f}$ are learned jointly by minimizing a suitable loss function (see Custom Loss Functions for Guided Training section). The masks $M^{\left(l\right)}$ enforce biological sparsity, reduce overfitting, and ensure each subnetwork aligns with known genotype–intermediate trait relationships.

Our BINN framework is highly modular: depending on the number, type, and interdependencies of available omics layers, the network can assume different connectivity patterns, ranging from single‐layer designs to staggered, stacked, or parallel architectures. A brief description of these variants is provided in the Supporting Information.

### Custom loss functions for guided training

BINNs are flexible and can be trained with any suitable loss function, ranging from conventional objectives to biologically informed criteria.

#### Standard mean squared error (MSE)

The simplest choice is the mean squared error between the true phenotype y and the prediction $\hat{y}$:

$$L_{\mathrm{MSE}}\left(y,\hat{y}\right)=\frac{1}{n}\sum_{i=1}^{n}{\left(y_{i}-{\hat{y}}_{i}\right)}^{2}.$$

#### Biologically informed soft‐constrained loss

To encourage the model's intermediate representations to align with measured omics traits, we add a soft constraint based on correlation. Let $T^{\left(l\right)}=\left[T_{1}^{\left(l\right)},\ldots ,T_{n}^{\left(l\right)}\right]$ be the predicted latent values at layer l and $Z^{\left(l\right)}=\left[Z_{1}^{\left(l\right)},\ldots ,Z_{n}^{\left(l\right)}\right]$ the corresponding ground‐truth intermediate measurements. Define the sample Pearson correlation

$$\rho^{\left(l\right)}=\frac{\sum_{i=1}^{n}\left(T_{i}^{\left(l\right)}-{\bar{T}}^{\left(l\right)}\right)\;\left(Z_{i}^{\left(l\right)}-{\bar{Z}}^{\left(l\right)}\right)}{\sqrt{\sum_{i=1}^{n}{\left(T_{i}^{\left(l\right)}-{\bar{T}}^{\left(l\right)}\right)}^{2}}\;\sqrt{\sum_{i=1}^{n}{\left(Z_{i}^{\left(l\right)}-{\bar{Z}}^{\left(l\right)}\right)}^{2}}},$$

where ${\bar{T}}^{\left(l\right)}$ and ${\bar{Z}}^{\left(l\right)}$ are the layerwise means. The biologically informed loss is then

$$L_{\mathrm{bio}}=L_{\mathrm{MSE}}\left(y,\hat{y}\right)+\lambda \sum_{l=1}^{L}\left[\;1-\rho^{\left(l\right)}\right],$$

where λ>0 is a hyperparameter balancing phenotype accuracy against intermediate‐trait alignment. One could instead enforce an exact match via an auxiliary hard‐constrained MSE $∥T^{\left(l\right)}-Z^{\left(l\right)}{∥}_{2}^{2}$, but the correlation‐based soft constraint is less restrictive, allows the model greater expressive power and proved more effective empirically in our analysis.

This biologically informed loss is optional: users may omit the correlation term (λ=0) to recover the standard MSE objective, or tune λ to any desired level of guidance.

### Transcriptomics‐derived BINN architecture summary

To provide a concrete instantiation of the general framework described above, we briefly summarize the architecture used for the maize TWAS application. The model is a single‐omics‐layer G2B2P network (L=1). SNP markers are selected via eQTL mapping of candidate genes identified through Elastic Net regression on gene expression data (L1 ratio=0.1; see Table S1). The binary mask $M^{\left(1\right)}$ connects each SNP to the gene(s) it was mapped to, routing marker information through gene‐level pathway subnetworks. Each subnetwork is a small fully connected network with a single hidden layer of 128 neurons and sigmoid activations, producing a single scalar latent variable per gene. These latent variables are concatenated and passed to a final integrator network with a single hidden layer of 128 neurons that predicts the flowering‐time phenotype. The specific number of input markers, selected genes, and detailed hyperparameter configurations for each trait and cross‐validation split are provided in Table S3.

### Sensitivity analysis for latent perturbations

To quantify the contribution of intermediate biological entities to the phenotype, we perform a *post hoc sensitivity analysis* across trained BINN ensembles. This approach assesses how controlled perturbations of individual latent variables influence the predicted phenotype, providing an interpretable measure of importance that generalizes across omics layers, phenotypes, and model instances.

#### Overview

Given a trained BINN ensemble consisting of S outer splits and F inner folds, let ${\left\{M_{s,f}\right\}}_{s=1..S,f=1..F}$ denote the set of trained models for a phenotype of interest. Each model $M_{s,f}$ contains intermediate latent representations $U^{\left(l\right)}$ at layer l, corresponding to biological entities such as genes, metabolites, or proteins. Sensitivity analysis probes each latent by replacing it with controlled constants and observing the resulting change in the model's mean predicted phenotype $\hat{y}$.

#### Step 1: Baseline computation

For each trained model, we first perform a forward pass on a held‐out test dataset to compute the baseline predicted phenotype ${\hat{y}}_{0}$ and the corresponding latent activations $U^{\left(l\right)}$. The mean $\mu_{j}$ and standard deviation $\sigma_{j}$ of each latent dimension $u_{j}^{\left(l\right)}$ are computed across all samples in the dataset.

#### Step 2: Latent clamping and phenotype response

To evaluate the sensitivity of entity j, we perform two controlled perturbations by *clamping* its latent activation to constant values across all samples:

$$u_{j}^{\left(l\right)}\left(\delta \right)=\mu_{j}+\delta \;\sigma_{j},\;\delta \in \left\{+a,-a\right\},$$

where a is the perturbation magnitude in units of standard deviation (we use a=1 throughout) while all other latents remain unaltered. The perturbed latent is then propagated through the trained model to produce new mean phenotype predictions ${\hat{y}}^{\left(+\right)}$ and ${\hat{y}}^{\left(-\right)}$. The per‐entity sensitivity under model $M_{s,f}$ is defined as the symmetric mean absolute deviation:

$$\Delta y_{j}^{\left(s,f\right)}=1/2\left(|{\hat{y}}^{\left(+\right)}-{\hat{y}}_{0}|,+,|{\hat{y}}^{\left(-\right)}-{\hat{y}}_{0}|\right),$$

capturing the magnitude of the phenotype's response to both upward and downward perturbations.

#### Step 3: Aggregation across models

Sensitivities are aggregated across all ensemble members that include entity j to obtain a robust importance estimate:

$${\bar{\Delta y}}_{j}=\frac{1}{|M\left(j\right)|}\sum_{\left(s,f\right)\in M\left(j\right)}\Delta y_{j}^{\left(s,f\right)},$$

where $M\left(j\right)$ is the subset of trained models that contain latent $u_{j}^{\left(l\right)}$. The resulting ${\bar{\Delta y}}_{j}$ represents the average change in predicted phenotype magnitude when the latent corresponding to entity j is clamped to values spanning its natural variability.

#### Step 4: Entity ranking and downstream analysis

Entities are ranked by their aggregated sensitivity ${\bar{\Delta y}}_{j}$, yielding a model‐based importance profile for the phenotype under study. This ranking can identify key intermediate traits, benchmark model interpretability against known associations, and guide downstream analyses such as candidate gene selection, pathway enrichment, or gene‐editing prioritization.

## CONFLICT OF INTEREST

None of the authors have a conflict of interest to disclose.

## Supporting information


**Table S1.** Assessment of the quality of the set of genes obtained using the ElasticNet models.
**Table S2.** Number of train, validation, and test lines for each subpopulation used in each fold (20%/80% train/test sparse data scenario).
**Table S3.** Number of genes selected per phenotype for the five data splits using *L*1 ratio=0.1 and total number of associated genetic markers derived from eQTL analysis.
**Figure S1.** Distribution of the phenotype time to bud outgrowth $y_{\mathrm{BO}}$, and intermediate traits auxin (A), sucrose (S), cytokinin (CK), and strigolactone (SL).
**Figure S2.** Distribution of the four phenotypes; days to anthesis and silking in NE and MI, for the real‐world maize TWAS dataset.
**Figure S3.** Test Spearman correlation distributions for prediction of days to Anthesis in NE and MI—comparing all the models (G2P on genotype, pink; B2P: Elastic Net on expression, green; G2B2P: BINN with network sparsity defined by lasso‐selected genes and eQTL‐selected markers, blue) across five independent 20%/80% train/test splits, each with fivefold cross‐validation.
**Figure S4.** Test Spearman correlation distributions for prediction of days to Silking in NE and MI—comparing all the models (G2P on genotype, pink; B2P: Elastic Net on expression, green; G2B2P: BINN with network sparsity defined by lasso‐selected genes and eQTL‐selected markers, blue) across five independent 20%/80% train/test splits, each with fivefold cross‐validation.
**Figure S5.** Test Spearman correlation distributions for (a) Silking NE, (b) Silking MI, and (c) Anthesis NE—comparing three predictive models (G2P: GBLUP on genotype, pink; B2P: Elastic Net on expression, green; G2B2P: BINN with expression‐informed sparsity, blue) across five independent 20%/80% train/test splits, each with fivefold cross‐validation.
**Figure S6.** Test Spearman correlation distributions for leave‐one‐population‐out experiments for (a) Silking NE, (b) Silking MI, and (c) Anthesis NE—comparing three predictive models (G2P: ridge regression on genotype, pink; B2P: Elastic Net on expression, green; G2B2P: BINN with expression‐informed sparsity, blue) across five independent 20% train splits, each with fivefold cross‐validation.
**Figure S7.** Test Spearman correlation distributions for four flowering‐time traits—(a) Anthesis NE, (b) Anthesis MI, (c) Silking NE, and (d) Silking MI—comparing three predictive models.
**Figure S8.** Test Spearman correlation distributions for four flowering‐time traits—(a) Anthesis NE, (b) Anthesis MI, (c) Silking NE, and (d) Silking MI—comparing three predictive models.
**Figure S9.** Flexible BINN architectures for diverse multi‐omics scenarios.

## Data Availability

All data generated for the shoot‐branching problem were produced in‐house by numerically integrating the ODE frameworks described in Powell et al. (2022) and Bertheloot et al. (2020). For the maize TWAS analyses, we used the publicly released genotype–expression–phenotype dataset from Torres‐Rodríguez et al. (2024), which is accessible via the original publication's data archive. The ElasticNet‐derived gene lists, trained BINN models, and accompanying inference notebook are provided to enable reproducibility of the results presented in this study and can be accessed via Zenodo under the DOI: 10.5281/zenodo.17477083. The full code used for training the models can be made available upon request through a Material Transfer Agreement (MTA) process.
