# Supplementary material for: Biology‐informed neural networks learn nonlinear representations from omics data to improve genomic prediction and biological discovery
Source: Plant J. 2026 Aug 11;127(3):e71076. doi: 10.1111/tpj.71076 (PMC13461207; doi:10.1111/tpj.71076)
Supplement: Supplementary file 1 [file TPJ-127-0-s001.docx]

Supplementary Materials for:
*Biology-informed neural networks learn nonlinear representations from omics data to improve genomic prediction and biological discovery*

Katiana Kontolati, Rini Jasmine Gladstone, Ian Davis, Ethan Pickering

# Overview

This document provides supplementary information supporting the findings and methodology of the main manuscript. It includes additional figures, tables, implementation details, and reproducibility notes for both the transcriptomics and metabolomics applications.

# Transcriptomics-derived BINN

## Introduction

Linking genetic variation to phenotypic traits remains a central challenge in plant genomics. Torres-Rodrı́guez et al. 2024, conducted a transcriptome‑wide association study (TWAS) on the Wisconsin Diversity Panel, consisting 693 temperate adapted maize inbred lines from seven heterotic pools: stiff stalk (SS), non-stiff stalk (NSS), iodent (IDT), tropical, popcorn, sweet corn, and other/unknown, to identify genes controlling flowering time. The authors collected full‑length RNA‑seq within a tight two‑hour window and recorded days to anthesis and silking at two Corn Belt locations, Nebraska and Michigan. From these data, they identified 21 genes exceeding stringent false‑discovery thresholds and mapped both *cis*‑ and *trans*‑eQTL, including loci in well‑characterized flowering regulators. The study’s power derives from its large sample size, precise sampling protocol, and deep sequencing depth, making it one of the most comprehensive TWAS datasets in maize.

Because this cohort provides the complete “genotype–expression–phenotype” triangle, it is ideal for evaluating our methodology. We hypothesize that embedding expression‑derived sparsity into the network architecture improves predictive accuracy over genotype‑only models, while still requiring only genotype data at inference. We constructed the BINN’s sparsity mask through a two‑step procedure. First, we trained an expression(biological domain knowledge)‑to‑phenotype (B2P) model using Elastic Net regression on the RNA‑seq data for each flowering trait, tuning the regularization parameters via cross‑validation. We then selected genes with non-zero coefficients. Second, we performed eQTL mapping on those candidate genes to identify their top SNP markers. The resulting SNP-gene pairs defined the binary mask connectivity matrix used to define our sparse genotype‑to‑expression‑to‑phenotype BINN architecture.

## Dataset and Preprocessing

We use the real-world maize data provided in Torres-Rodriguez et al. 2024 , which includes flowering times (days to silking and anthesis) measured at two field locations (Nebraska and Michigan), RNA-seq gene expression data from leaf samples, and genotypes from 15.8 million single nucleotide polymorphism (SNP) markers. The dataset consists of 690 lines from the Wisconsin Diversity Panel, covering 7 heterotic groups (stiff stalk, non-stiff stalk, iodent, tropical, popcorn, sweet corn, and other/unknown) and expression values of 39,756 genes. We followed the data pre-processing steps described in Torres-Rodriguez et al. and used the spatially-corrected flowering time values that were provided. Fig. 2 shows the distribution of the 4 phenotypes across all the 690 lines. We also dropped the unexpressed genes, with median TPM less than 0.01, from further analysis. This reduced the total number of genes to 24,874. Furthermore, markers with a minor allele frequency (MAF) below 5% were dropped, and PLINK (Purcell et al. 2007) was used to prune highly correlated markers (--indep-pairwise 1000 500 0.5), leaving 3.2 million markers for further analysis. The pre-processed expression and marker data is, then, used for feature selection for the BINN model.

### Feature Selection

The BINN architecture is sparsified by selecting a subset of genes and markers through a biologically-informed process. The genes are selected using a linear elastic net model and markers are selected through eQTL process. This reduces the input features (markers) by 2 orders of magnitude and the length of intermediate layer (genes) by 1 order of magnitude.

1. **Gene selection :** To identify a reduced set of significant genes influencing flowering time, we developed ElasticNet models utilizing expression data from 24,874 genes as predictors, with days to silking and anthesis for NE and MI (four distinct models) as the response variables. The expression data, measured in Transcripts Per Million (TPM), was normalized to a range of 0 to 2, following the methodology outlined by Torres-Rodriguez et al. The ElasticNet models were implemented using Scikit-Learn’s ElasticNetCV (Kramer and Kramer 2016), and we selected the number of genes based by varying the values of the L1 ratio by subsetting the features with non-zero coefficients. We used 5-fold cross-validation for model building. To assess the quality of the selected genes, we compared the overlap between genes identified as influencing flowering time in Torres-Rodriguez et al. and those selected by the ElasticNet models. Table 1 presents the number of genes identified for various values of the L1 ratio, ($l$), and the corresponding percentage of overlap with the flowering time genes reported in Torres-Rodriguez et al., based on models trained using expression values from all the 690 lines. This analysis demonstrates that as the L1 ratio decreases, the number of selected genes increases, and a lower L1 ratio facilitates the identification of a greater number of significant genes.

Table 1. Assessment of the quality of the set of genes obtained using the ElasticNet models

| Phenotype | # of significant  flowering time  genes from Torres-Rodriguez et al. | # of genes from  ElasticNet models | | | | | % overlap of  significant genes | | | |
| --- | --- | --- | --- | --- | --- | --- | --- | --- | --- | --- |
|  | | $l=1.0$ | $l=0.5$ | $l=0.2$ | | $l=0.1$ | $l=1.0$ | $l=0.5$ | $l=0.2$ | $l=0.1$ |
| Days to Silking, MI | 17 | 317 | 568 | | 970 | 1,662 | 70 | 82 | 100 | 100 |
| Days to Anthesis, MI | 16 | 320 | 412 | | 816 | 1,467 | 69 | 81 | 94 | 100 |
| Days to Silking, NE | 16 | 234 | 413 | | 756 | 1,378 | 69 | 81 | 100 | 100 |
| Days to Anthesis, NE | 14 | 259 | 383 | | 760 | 1,328 | 64 | 100 | 100 | 100 |

2. **Marker selection :** For each gene selected through the ElasticNet model, significant markers were identified using eQTL analysis (Holloway et al. 2011) as outlined in Torres-Rodriguez et al., using mixed linear model (Zhang et al. 2010) in rMVP (Yin et al. 2021) and including three principal components as covariates. For all the identified genes, the top 20 statistically significant markers are selected for training the BINN models.

Figure 2. Distribution of the four phenotypes; days to anthesis and silking in NE and MI, for the real-world maize TWAS dataset

## Model Architecture

The BINN architecture comprises of three layers: an input layer consisting of the reduced set of markers, an intermediate layer in which each node represents a gene, and a final output layer that generates the predicted phenotype values. Every node (gene) in the intermediate layer is connected to 20 eQTL-selected nodes (significant markers) from the input layer via a pathway sub-network, which is structured as a fully connected network (FCN) with a single hidden layer of dimension, $H=128$, and a sigmoid activation function. Note that certain markers may be mapped to more than one gene through the eQTL mapping procedure; consequently, some markers participate in multiple pathway sub-networks simultaneously. The outputs from all the pathway sub-networks are concatenated and subsequently fed into another FCN, with a single hidden layer of dimension, $F=128$, to predict the flowering time, $y_{\text{FT}}$.

## Experimental Setup

The experiments were designed to evaluate the performance of the BINN under conditions of sparse data. Consequently, all models were trained on 20% of the dataset while being tested on the remaining 80%. The entire dataset was divided into five non-overlapping subsets (outer splits), while ensuring that the lines from all heterotic groups were represented in the same proportion as the full dataset. The exact number of train, validation and test lines used in each independent fold is shown in Table 2.

**Inner cross-validation (BINN only)**. For each outer split, the BINN models were further trained and evaluated using an inner 5-fold cross-validation within the 20% training partition. This inner CV was used for hyperparameter tuning and model selection, resulting in five trained BINN models per outer split and 25 BINN models in total across all outer splits. Each of these 25 models was then evaluated on the fixed 80% held-out test set from its corresponding outer split. For the GBLUP baseline, no inner cross-validation was performed, as the model has a closed-form solution with the regularization parameter determined by the ratio of variance components. This results in a single GBLUP model per outer split and five GBLUP models in total.

**Random split experiments**. In the random split experiments (Figures 2b, 2c of main text), each of the five outer splits serves as an independent replicate. For BINN, the inner 5-fold CV within each outer split produces five models per split, yielding 25 prediction accuracy values in total. For GBLUP, a single model is trained per outer split, yielding five prediction accuracy values in total. All models within a given outer split are evaluated on the same fixed 80% held-out test set, and Spearman rank correlations are computed both across all pooled lines and within each heterotic subpopulation.

**Leave-one-population-out experiments**. For the leave-one-population-out experiments (Figure 2d of main manuscript), all lines from a given population were held out as a fixed test set. From the remaining lines, we created five distinct 20/80 train/validation splits following the same protocol as described above, with BINN models undergoing inner 5-fold CV within each training partition and GBLUP models trained once per split. This is consistent with our emphasis on evaluating models under sparse-data conditions, as training on only 20% of the remaining lines after removing an entire population reflects the low-data regimes most relevant to practical breeding scenarios.

**Feature selection and data leakage prevention**. To prevent any data leakage during feature selection, gene and marker selection were performed independently for each outer split, ensuring that the corresponding features were utilized exclusively when training the model on that specific subset of data. For gene selection, we used an L1 ratio of $l_{1}=0.1$ for the Elastic Net models, corresponding to the best-performing setting identified in Table 1 of the main text (bolded entry). Gene selection was performed with 5-fold cross-validation. Table 3 presents the number of selected genes and the corresponding number of unique input markers per phenotype across all data splits. For every selected gene, we selected 20 markers based on the eQTL analysis. It is important to note that expression data was used solely for feature selection to introduce sparsity in the network architecture and was not utilized for model training. Therefore, only genotype data is required for training and validation of the model.

**BINN training details**. We trained all the BINN models with the Adam optimizer with an initial learning rate $\alpha={10}^{-3}$ and batch size of 16. Training was terminated if the validation loss did not improve for 20 consecutive epochs, with a maximum limit of 500 epochs. As BINN is a neural network, it necessitates a validation dataset during training. However, the baseline models utilize the entire 20% of the data for training without requiring a validation set. To ensure a fair comparison between the models, we initially trained the BINN models using 16% of the data for training and 4% for early stopping/validation for each of the five inner CV folds. We recorded the epoch that yielded the best model performance for each of these runs and subsequently retrained the BINN model using the full 20% of the data for the same number of epochs without a validation set.

Table 2. Number of train, validation and test lines for each subpopulation used in each fold (20%/80% train/test sparse data scenario).

| **Population** | **Train** | **Validation** | **Test** |
| --- | --- | --- | --- |
| All | 65 | 65 | 560 |
| Others | 29 | 29 | 241 |
| SS | 15 | 15 | 129 |
| NSS | 9 | 9 | 76 |
| IDT | 5 | 5 | 47 |
| Tropical | 3 | 3 | 26 |
| Popcorn | 2 | 2 | 18 |
| Sweet corn | 2 | 2 | 23 |

Table 3. Number of genes selected per phenotype for the five data splits using $l=0.1$ and total number of associated genetic markers derived from eQTL analysis.

| *Data subset* | *Days to silking, MI* | | *Days to anthesis, MI* | | *Days to silking, NE* | | *Days to anthesis, NE* | |
| --- | --- | --- | --- | --- | --- | --- | --- | --- |
|  | genes | markers | genes | markers | genes | markers | genes | markers |
| *Split 1* | 1,271 | 24,070 | 1,041 | 19,934 | 1,010 | 19,135 | 933 | 17,779 |
| *Split 2* | 1,230 | 23,307 | 995 | 19,114 | 1,071 | 20,339 | 912 | 17,492 |
| *Split 3* | 1,079 | 20,497 | 919 | 17,349 | 969 | 18,378 | 941 | 17,895 |
| *Split 4* | 1,188 | 22,862 | 985 | 18,957 | 1,044 | 20,058 | 848 | 16,363 |
| *Split 5* | 1,235 | 23,648 | 1,018 | 19,611 | 1,042 | 20,032 | 933 | 17,921 |

## Baseline Model

In the main manuscript, we compare the performance of the BINN models against benchmark genotype-to-phenotype (G2P) and domain expression-to-phenotype (B2P) models, all of which are linear in nature. For G2P, we trained GBLUP and ridge regression models, whereas, for B2P, a ridge regression model was trained. All the ridge regression models were implemented using Scikit-Learn’s (Kramer and Kramer 2016) RidgeCV function with 3-fold cross-validation. We used the GBLUP implementation from BGLR (Pérez and Los Campos 2014) with 5000 iterations after 1000 burn-in iterations.

The G2P models were trained using every 100th marker, as we found no significant difference in performance compared to using all 3.2 million markers. On the other hand, B2P model was trained on the expression (in TPM) of all 24,874 genes. Prior to model training, the TPM values were transformed using the Box-Cox normalization with a parameter $\lambda=0.0282$ to approximate a normal distribution.

## Evaluation Strategy

The performance of the BINN models is compared with that of the baseline models using Spearman's rank correlation coefficients (Myers and Sirois 2004). Specifically, we calculated the correlation between the predicted and observed phenotype values on the 80% held-out test set. For the random split experiments, correlation values are computed for each of the 25 BINN models (five inner CV folds × five outer splits) and each of the five GBLUP models (one per outer split), as well as within each heterotic group represented in the dataset. For the leave-one-population-out experiments, correlations are computed analogously, with the held-out population serving as the fixed test set. It is important to note that since all models within a given outer split are evaluated on the same fixed 80% held-out lines, this may underestimate the true model variance, potentially resulting in less variability in our test metrics.

For the statistical comparison, we treated each heterotic group as an independent trial, aggregating Spearman correlations across all inner CV folds, outer splits, and all four phenotypes, and then applied a paired t-test (Hsu and Lachenbruch 2014) to determine whether the BINN model's performance is significantly better than that of the G2P baseline. In the results reported in the main text we obtained $p=2.23\times{10}^{-6}$, well below the 0.05 significance threshold, allowing us to reject the null hypothesis and conclude that BINN significantly outperforms the baseline model in the majority of comparisons.

## Additional Evaluation Results

In this section, we discuss additional experiments conducted to further evaluate the impact of biologically informed feature selection and network sparsity in BINNs. First, we compare the performance of the BINN model against various additional baseline models. Specifically, we consider four G2P baseline models – GBLUP (shown in Figure 2 of the main manuscript), GBLUP using “bio-informed” (eQTL-selected) markers, Ridge Regression using every 100th marker and Ridge Regression using eQTL-selected markers and two B2P models - Ridge Regression using all the genes (shown in Figure 2 of the main manuscript) and Ridge Regression using “bio-informed” ElasticNet-selected genes. Figures 3 and 4 illustrate the performance of these models in predicting four flowering time traits — days to anthesis and silking in both NE and MI. We found that, biologically informed markers and genes enhance the model performance regardless of the baseline models, as evidenced in Figures 3 and 4. These results underscore our finding that biologically informed sparsity imparts essential inductive bias to the model, thereby, improving its predictive accuracy.

|  |  |
| --- | --- |

Figure 3. Test Spearman correlation distributions for prediction of days to Anthesis in NE and MI — comparing all the models (G2P on genotype, pink; B2P: Elastic Net on expression, green; G2B2P: BINN with network sparsity defined by lasso selected genes and eQTL-selected markers, blue) across five independent 20%/80% train/test splits, each with five‑fold cross‑validation. Results of four G2P models are shown - GBLUP (G2P-GBLUP), GBLUP on bio-informed (eQTL-selected) markers (GBLUP-BI), Ridge Regression on every 100th marker (G2P-RR) and Ridge Regression on bio-informed markers (G2P-RR-BI). Additionally, results of two B2P models are also shown - Ridge Regression on all the genes (B2P-RR) and Ridge Regression on bio-informed (ElasticNet-selected) genes (B2P-RR-BI). Each subplot shows results for all lines pooled and for each of the seven distinct subpopulations.

|  |  |
| --- | --- |

Figure 4. Test Spearman correlation distributions for prediction of days to Silking in NE and MI — comparing all the models (G2P on genotype, pink; B2P: Elastic Net on expression, green; G2B2P: BINN with network sparsity defined by lasso selected genes and eQTL-selected markers, blue) across five independent 20%/80% train/test splits, each with five‑fold cross‑validation. Results of four G2P models are shown - GBLUP (G2P-GBLUP), ), GBLUP on bio-informed (eQTL-selected) markers (GBLUP-BI), Ridge Regression on every 100th marker (G2P-RR) and Ridge Regression on bio-informed markers (G2P-RR-BI). Additionally, results of two B2P models are also shown - Ridge Regression on all the genes (B2P-RR) and Ridge Regression on bio-informed (ElasticNet-selected) genes (B2P-RR-BI). Each subplot shows results for all lines pooled and for each of the seven distinct subpopulations.

Figure 5. Test Spearman correlation distributions for (a) Silking NE, (b) Silking MI and (c) Anthesis NE - comparing three predictive models (G2P: GBLUP on genotype, pink; B2P: Elastic Net on expression, green; G2B2P: BINN with expression‑informed sparsity, blue) across five independent 20%/80% train/test splits, each with five‑fold cross‑validation. Each subplot shows results for all lines pooled (“all”) and for each of the seven distinct subpopulations (SS, NSS, IDT, Popcorn, Sweet corn, Tropical and Others). The pink dashed line marks the median of the G2P baseline; the dashed line for G2B2P is colored green when its median exceeds that baseline or red when it falls below. Legends report the median percent change of G2B2P relative to G2P.

Figure 6. Test Spearman correlation distributions for leave-one-population-out experiments for (a) Silking NE, (b) Silking MI and (c) Anthesis NE - comparing three predictive models (G2P: Ridge Regression on genotype, pink; B2P: Elastic Net on expression, green; G2B2P: BINN with expression‑informed sparsity, blue) across five independent 20% train splits, each with five‑fold cross‑validation.Each subplot shows results for the predictions on the six distinct subpopulations (SS, NSS, IDT, Popcorn, Sweet corn, and Tropical) using the models that are trained by **leaving out** the corresponding subpopulation from the training data. The pink dashed line marks the median of the G2P baseline; the dashed line for G2B2P is colored green when its median exceeds that baseline or red when it falls below. Legends report the median percent change of G2B2P relative to G2P.

Furthermore, we conducted an ablation study to evaluate the impact of biologically informed network sparsity on the performance of the BINN model. This involved incorporating randomly selected network sparsity into the BINN architecture and comparing its predictive performance with that of the baseline models. Figure 7 presents the results for the BINN model, where network sparsity is defined by randomly selected genes and eQTL-selected markers. To ensure a fair evaluation, we maintained an equal number of randomly selected genes as in the biologically informed case. Figure 8 displays similar results for network sparsity defined by randomly selected genes and markers.

Figure 7. Test Spearman correlation distributions for four flowering‑time traits—(a) Anthesis NE, (b) Anthesis MI, (c) Silking NE, and (d) Silking MI—comparing three predictive models. Here, the network sparsity of G2B2P (BINN) model is defined by **randomly selected genes** and **eQTL-selected markers**, blue). Models were trained across five independent 20%/80% train/test splits, each with five‑fold cross‑validation. Each subplot shows results for all lines pooled and for each of the seven distinct subpopulations.

Figure 8. Test Spearman correlation distributions for four flowering‑time traits—(a) Anthesis NE, (b) Anthesis MI, (c) Silking NE, and (d) Silking MI—comparing three predictive models. Here, the network sparsity of G2B2P (BINN) model is defined by **randomly selected genes** and **randomly selected markers**, blue). Models were trained across five independent 20%/80% train/test splits, each with five‑fold cross‑validation. Each subplot shows results for all lines pooled and for each of the seven distinct subpopulations.

Our observations from these analyses indicate that as the level of random connections in the network architecture increases—from randomly selected genes to randomly selected genes and markers—the performance of the BINN model significantly declines.

# Metabolomics-derived BINN

Axillary bud outgrowth is the key developmental process driving shoot branching in plants. Its regulation arises from an intricate network of hormonal and sugar signals. In the framework of Bertheloot et al. (Bertheloot et al. 2020), auxin (A) concentration produced at the shoot apex is transported basipetally and inhibits cytokinin (CK) synthesis while promoting strigolactone (SL) production. Cytokinin levels and strigolactone levels then act antagonistically to activate or repress bud growth, respectively, and sucrose availability (S) acts as an enabling cue. Bertheloot et al. showed that this minimal network captures the switch-like behavior of bud outgrowth across species and can be approximated as a system of ordinary differential equations (ODEs) (Bertheloot et al. 2020)). Powell et al. (Powell et al. 2022) then extended this ODE framework by parameterizing each interaction term and intermediate-trait (hormones and sucrose) level with additive genetic values calculated based on the additive genetic effects at multiple causal loci.

The introduction of genetic variation into the shoot‐branching network yields a fully specified G2P model, enabling in silico selection experiments that reveal how interactions among intermediate traits drive selection response, precisely the process breeders seek to optimize. Because this framework integrates genotype, metabolite levels, and phenotype, it functions as a biologically‐informed model, embedding known genetic loci-metabolite relationships as inductive biases. Moreover, having access to the exact ODEs used to generate synthetic data gives us a ground‐truth baseline against which to develop and rigorously benchmark our BINN architectures. Although the mapping from genotype to metabolite in this system is exactly specified, in real breeding populations we typically know only statistical associations and never the true functional forms. We therefore hypothesize that a BINN can recover these hidden relationships, learning the underlying network dynamics directly from data and providing both accurate predictions and mechanistic insight.

## Adapted System of Equations

We generate a large synthetic dataset following the ODE‐based shoot‐branching network of Bertheloot et al. (Bertheloot et al. 2020) extended by Powell et al. (Powell et al. 2022). For reproducibility we outline the exact set of equations below. Given $N$ lines and $L$ causal loci per pathway, the procedure is:

#### 1. Simulate additive genetic effects.

Draw locus effects $\{\beta_{j}{\}}_{j=1}^{4L}$ from a standard normal distribution. Denote the resulting effect vector by

$$\boldsymbol{\beta}=(\beta_{1},\ldots,\beta_{4L})^{\top}.$$

#### 2. Generate genotypes.

Form the genotype matrix

$$G\in\{0,1{\}}^{N\times4L}, G_{i,j}\sim Bernoulli(0.5),$$

where row $i$ represents line $i$ and columns $1\ldots L$, $L+1\ldots2L$, $2L+1\ldots3L$, $3L+1\ldots4L$ correspond to auxin (A), sugar (S), cytokinin (CK), and strigolactone (SL) pathways.

#### 3. Compute additive pathway values.

For each pathway $p\in\{A,S,CK,SL\}$, let $\boldsymbol{\beta}^{(p)}$ and $G^{(p)}$ denote the corresponding length-$L$ subvector and submatrix. The raw genetic value for pathway $p$ in line $i$ is

Equation 1

$$g_{i}^{(p)} = \sum_{j=1}^{L} G_{i,j}^{(p)} \beta_{j}^{(p)}.$$

#### 4. Rescale to steady‐state trait levels.

Powell et al. (2022) rescale genetic values by dividing by the theoretical maximum (sum of 2 times all locus effects). As L grows, no individual approaches this theoretical maximum under Bernoulli(0.5) genotypes, so that normalization compresses all values into a narrow band with minimal variation. We instead normalize by the empirical population maximum, then apply pathway-specific scale and offset parameters to produce intermediate trait ranges comparable to those in Powell et al. (2022):

Equation 2

$$x_{i}^{(p)}=\left( \frac{g_{i}^{(p)}}{2 \max_{i}|g_{i}^{(p)}|}+1/2 \right)s_{p}+b_{p},$$

with pathway‐specific scale $s_{p}$ and offset $b_{p}$:

$$(s_{A},b_{A})=(2.5,0), (s_{S},b_{S})=(2.4,0.1), (s_{\mathrm{CK}},b_{\mathrm{CK}})=(0.6,0.4), (s_{\mathrm{SL}},b_{\mathrm{SL}})=(0.9,0.2).$$

#### 5. Compute interaction terms and intermediate traits.

After computing raw genetic values $g^{(p)}$ via Equation 1, we first linearly map auxin and sugar to obtain

Equation 3

$$\begin{aligned} x_{i}^{(A)}&=g_{i}^{\left( A \right)}, &x_{i}^{(S)}&=g_{i}^{(S)}. \end{aligned}$$

Cytokinin and strigolactone receive additional interaction adjustments as:

Equation 4

$$\begin{aligned} x_{i}^{(CK)}&=\frac{g_{i}^{(CK)} \gamma_{CK\leftarrow A}^{(i)} + \gamma_{CK\leftarrow S}^{(i)}}{0.99}, \\ x_{i}^{(SL)}&=\frac{g_{i}^{(SL)} + \gamma_{SL\leftarrow A}^{(i)}}{0.86}. \end{aligned}$$

where $\gamma_{CK\leftarrow A}^{(i)}$, $\gamma_{CK\leftarrow S}^{(i)}$, and $\gamma_{SL\leftarrow A}^{(i)}$ are computed as

Equation 5

$$\begin{aligned} \gamma_{CK\leftarrow A}^{(i)}&=\frac{1}{1+0.96 x_{i}^{(A)}},&\gamma_{CK\leftarrow S}^{(i)}&=\frac{0.25 (x_{i}^{(S)})^{2}}{0.19+(x_{i}^{(S)})^{2}}, \\ \gamma_{SL\leftarrow A}^{(i)}&=\frac{24.89 (x_{i}^{(A)})^{2}}{294.58+(x_{i}^{(A)})^{2}}. \end{aligned}$$

#### 6. Integrator and phenotype.

The intermediate traits are passed through the signal integrator term as

Equation 6

$$\begin{aligned} \gamma_{I\leftarrow CK}^{(i)}&=\frac{287.53}{1+1000 x_{i}^{\left( \mathrm{CK} \right)^{2}}}, \\ \gamma_{I\leftarrow SL,S}^{(i)}&=5.64 \frac{(x_{i}^{(SL)})^{2}}{1+\left( 0.00418+7.10 (x_{i}^{(S)})^{2} \right) (x_{i}^{(SL)})^{2}}. \end{aligned}$$

The composite integrator signal for line $i$ is

Equation 7

$$I_{i}=0.33 + \gamma_{I\leftarrow CK}^{(i)} + \gamma_{I\leftarrow SL,S}^{(i)}.$$

Time to bud outgrowth is then obtained by a population-anchored linear transformation with an upper cap:

Equation 8

$$y_{BO,i}=min\{-3.5 \min_{j}I_{j}+3.5 I_{i}, 8.3\},$$

and lines with $y_{BO,i}<8.3$ are scored as having initiated bud outgrowth.

#### Because our normalization (Equation 2) uses the empirical rather than theoretical maximum, the integrator values $I_{i}$ occupy a different range than in Powell et al. (2022), and their original fixed intercept (-2.2) can yield negative bud outgrowth times under our rescaling. Replacing it with $-3.5 \min_{j}I_{j}$ anchors the minimum bud outgrowth time at zero while preserving the original sensitivity (3.5 day per mol) and upper cap (8.3 days). Since $\min_{j}I_{j}$ is a fixed scalar computed once during data generation, this does not introduce inter-individual dependencies.

#### 7. Final dataset.

We generate the genotype matrix $X=G$, the phenotype vector $y_{\mathrm{BO}}$, and the true intermediate traits $\{x^{(A)},x^{(S)},x^{(CK)},x^{(SL)}\}$ for downstream model training and evaluation.

## Data Generation

We generated a synthetic dataset using the equations outlined in the Adapted System of Equations section sing our own Python implementation. We consider $L=400$ causal loci per pathway which results in $1,600$ loci in total. A large dataset of $N=100,000$ lines was generated from which $10\%$ was used for validation and $20\%$ for testing fixed across models. The remaining $70\%$ was used to randomly sample training subsets used in the experiments described below. Below, we show the distribution of the phenotype $y_{\text{BO}}$ and the four intermediate traits (A,S,CK,SL).

Figure 1. Distribution of the phenotype time to bud outgrowth $y_{\text{BO}}$, and intermediate traits auxin (A), sucrose (S), cytokinin (CK) and strigolactone (SL).

## Model Architecture

Each pathway subnetwork is a shallow fully‐connected network (FCN) with hidden dimension $H=32$, inter‐layer sigmoid activations that aim to emulate the Hill-like dynamics of the shoot-branching network, and a one‐dimensional output. The four pathway outputs are concatenated and passed through a final FCN with hidden dimension $F=16$ to predict $y_{BO}$. Any SNPs not assigned to a pathway are fed into a residual FCN of the same size.

## Experimental Setup

All BINN experiments used the same training pipeline, implemented in PyTorch (Paszke et al. 2019). Below we summarize the key hyperparameters and procedures:

#### Data splits and noise injection.

For each run, we randomly sample from the full set $N_{train}$ lines for training, $N_{val}=0.1\times N_{total}$ for validation, and $N_{test}=0.2\times N_{total}$ for testing. To test the robustness of model to noisy data we then add Gaussian noise to the targets:

$$y_{i}\leftarrow y_{i} \left( 1+\epsilon_{i}^{(y)} \right), \epsilon_{i}^{(y)}\mathcal{\sim N(}0,\sigma_{y}^{2}),$$

and to each intermediate trait $p\in\{A,S,CK,SL\}$:

$$x_{i}^{(p)}\leftarrow x_{i}^{(p)} \left( 1+\epsilon_{i}^{(p)} \right), \epsilon_{i}^{(p)}\mathcal{\sim N(}0,\sigma_{inter}^{2}),$$

where $\sigma_{y}=10\%$ and $\sigma_{inter}=5\%$.

#### Optimization.

We trained each BINN with:

- **Optimizer:** Adam, initial learning rate $\alpha={10}^{-3}$, no weight decay.
- **Batch size:** 64.
- **Scheduler:** ReduceLROnPlateau on the validation loss, factor 0.5, patience 20 epochs.
- **Early stopping:** terminate if validation loss does not improve for 20 consecutive epochs, up to a maximum of 500 epochs.

#### Repetitions, dataset sizes, and random seeds.

We evaluated each BINN configuration over nine logarithmically spaced training set sizes ($500$ to $20,000$ samples), combined with varying noise levels and intermediate‐trait availability. For each of these dataset sizes, we performed $n=5$ independent runs using different random seeds. Genotypes were drawn once per size, and each seed controlled the train/validation/test split (80/10/10), the network weight initialization, and the Gaussian noise added to both the final phenotype and intermediate traits.

## Baseline Models

To benchmark the performance of our BINN architectures, we compared against two standard models trained on the same synthetic data using our own implementation:

#### Ridge regression.

We fit a linear model

$$\hat{y}=Xw+b,$$

with L${}^{2}$ regularization on the weights. Concretely, the model implements

$$\mathcal{L(}w,b) = \frac{1}{N}\sum_{i=1}^{N} (y_{i}-(x_{i}^{\top}w+b))^{2} + \lambda\parallel w\parallel_{2}^{2},$$

where $x_{i}\in\mathbb{R}^{p}$ is the genotype vector, $y_{i}$ the target, and $\lambda=0.01$ the regularization coefficient. Training proceeds by minimizing this loss via AdamW with the same learning rate and early‐stopping schedule as the BINNs.

#### Fully‐connected network (FCN).

As a representative “black‐box” nonlinear model, we trained a small deep network with two hidden layers of size 64 and ReLU activations:

$$\begin{aligned} h^{(1)}&=ReLU(W^{(1)}x+b^{(1)}), \\ h^{(2)}&=ReLU(W^{(2)}h^{(1)}+b^{(2)}), \\ \hat{y}&=W^{(3)}h^{(2)}+b^{(3)}. \end{aligned}$$

The model parameters $\{W^{(k)},b^{(k)}\}$ are learned by minimizing mean‐squared error:

$$\mathcal{L=}\frac{1}{N}\sum_{i=1}^{N} (y_{i}-\hat{y}_{i})^{2},$$

using the same optimizer, learning rate, batch size, and early‐stopping criteria as for the BINNs.

## Evaluation Metric

To quantify predictive performance, we compute the mean squared error (MSE) on the held‐out test set for each model, dataset size, and random seed. Specifically, for each dataset size $N_{train}$, noise configuration, and intermediate‐trait fraction, we perform $n=5$ independent runs yielding test errors

$$\mathrm{MSE}_{r}=\frac{1}{N_{test}}\sum_{i=1}^{N_{test}} (y_{i}-\hat{y}_{i}^{(r)})^{2}, r=1,\ldots,n.$$

We then summarize these $5$ values via their mean and standard deviation in the main text, and visualize their full distribution using boxplots.

# Alternative BINN Architectures

In the main text, we described our canonical BINN design and its instantiation for (1) the synthetic shoot‐branching problem and (2) the maize TWAS dataset. The following examples are not exhaustive alternatives but illustrate the flexibility of our architecture in handling diverse multi‑omics contexts:

Figure 9. Flexible BINN architectures for diverse multi‑omics scenarios. (a) Single‑layer BINN: A single intermediate omics layer (e.g. gene expression) feeds directly into the final integrator; this configuration was used for the TWAS problem. (b) Staggered‑layer BINN: Within a single omics modality, pathway subnetworks exchange information via sparse cross‑connections. For example, in the shoot‑branching network, both auxin (A) and sucrose (S) modules feed into the cytokinin (CK) and strigolactone (SL) subnetworks before final integration.(c) Stacked‑layer BINN: Multiple omics layers are embedded in series - outputs from the first layer’s subnetworks become inputs to the second layer’s modules - mirroring cascades such as transcriptome → metabolome. (d) Parallel‑layer BINN: Independent subnetworks process each omics type (e.g. transcriptomics, proteomics, methylation) in parallel, with their latent outputs concatenated only at the final integrator (“late fusion”).

## Single‐layer BINN

This model - used for the TWAS problem - has one intermediate omics layer (gene expression) feeding directly into the final integrator. It is appropriate when a single data type dominates predictive power, as in transcriptome‑wide association studies without the availability of additional -omics data such metabolomics or proteomics.

## Staggered‐layer BINN

In cases with a single omics modality but with rich intra‐layer dependencies, we allow pathway subnetworks to exchange information via sparse cross‐connections. For example, in the shoot‐branching network, auxin (A) and sucrose (S) subnetworks both feed into the cytokinin (CK) and strigolactone (SL) modules. These staggered links capture non‐hierarchical, within‐layer interactions, such as gene–gene or metabolite–metabolite cross‐talk, before passing all signals to the final integrator.

## Stacked‐layer BINN

When multiple omics layers form a biological cascade, such as genotype → transcriptome → metabolome, we embed each layer as a separate set of subnetworks connected in series. The latent outputs of the first layer become the inputs to the second layer’s pathway modules, and so on, before final integration. This design mirrors natural hierarchies (e.g. expression driving metabolite synthesis) and is appropriate whenever upstream molecular changes are known to mediate downstream effects.

## Parallel‑layer BINN

Each omics type (e.g. transcriptomics, proteomics, methylation) is processed by an independent subnetwork, and their latent outputs are concatenated only at the final integrator. This “late fusion” design suits orthogonal assays with minimal direct cross‑talk, such as combining methylation and ATAC‑seq to predict complex agronomic traits.

All variants share the same core elements, biological sparsity masks, shallow FCNs per module, and a unified integrator, yet differ in how modules interconnect, allowing users to tailor the model to their study’s data structure and biological assumptions.

# References

Bertheloot, Jessica, François Barbier, Frédéric Boudon, et al. 2020. “Sugar Availability Suppresses the Auxin-Induced Strigolactone Pathway to Promote Bud Outgrowth.” *New Phytologist* 225 (2): 866–79.

Holloway, Beth, Stanley Luck, Mary Beatty, J-Antoni Rafalski, and Bailin Li. 2011. “Genome-Wide Expression Quantitative Trait Loci (eQTL) Analysis in Maize.” *BMC Genomics* 12: 1–14.

Hsu, Henry, and Peter A Lachenbruch. 2014. “Paired t Test.” *Wiley StatsRef: Statistics Reference Online*.

Kramer, Oliver, and Oliver Kramer. 2016. “Scikit-Learn.” *Machine Learning for Evolution Strategies*, 45–53.

Myers, Leann, and Maria J Sirois. 2004. “Spearman Correlation Coefficients, Differences Between.” *Encyclopedia of Statistical Sciences* 12.

Paszke, Adam, Sam Gross, Francisco Massa, et al. 2019. “PyTorch: An Imperative Style, High-Performance Deep Learning Library.” *Advances in Neural Information Processing Systems* 32. <https://pytorch.org/>.

Pérez, Paulino, and Gustavo de Los Campos. 2014. “Genome-Wide Regression and Prediction with the BGLR Statistical Package.” *Genetics* 198 (2): 483–95.

Powell, Owen M, Francois Barbier, Kai P Voss-Fels, Christine Beveridge, and Mark Cooper. 2022. “Investigations into the Emergent Properties of Gene-to-Phenotype Networks Across Cycles of Selection: A Case Study of Shoot Branching in Plants.” *In Silico Plants* 4 (1): diac006.

Purcell, Shaun, Benjamin Neale, Kathe Todd-Brown, et al. 2007. “PLINK: A Tool Set for Whole-Genome Association and Population-Based Linkage Analyses.” *The American Journal of Human Genetics* 81 (3): 559–75.

Torres-Rodrı́guez, J Vladimir, Delin Li, Jonathan Turkus, et al. 2024. “Population-Level Gene Expression Can Repeatedly Link Genes to Functions in Maize.” *The Plant Journal* 119 (2): 844–60.

Yin, Lilin, Haohao Zhang, Zhenshuang Tang, et al. 2021. “rMVP: A Memory-Efficient, Visualization-Enhanced, and Parallel-Accelerated Tool for Genome-Wide Association Study.” *Genomics, Proteomics & Bioinformatics* 19 (4): 619–28.

Zhang, Zhiwu, Elhan Ersoz, Chao-Qiang Lai, et al. 2010. “Mixed Linear Model Approach Adapted for Genome-Wide Association Studies.” *Nature Genetics* 42 (4): 355–60.
